# Phylogeny and expression of carbonic anhydrase-related proteins

**DOI:** 10.1186/1471-2199-11-25

**Published:** 2010-03-31

**Authors:** Ashok Aspatwar, Martti EE Tolvanen, Seppo Parkkila

**Affiliations:** 1 Bioinformatics Group, Institute of Medical Technology, 33014 University of Tampere, Tampere, Finland; 2 Institute of Medical Technology and School of Medicine, University of Tampere, 33520 Tampere, Finland; 3 Centre for Laboratory Medicine, Tampere University Hospital, 33520 Tampere, Finland

## Abstract

**Background:**

Carbonic anhydrases (CAs) are found in many organisms, in which they contribute to several important biological processes. The vertebrate α-CA family consists of 16 subfamilies, three of which (VIII, X and XI) consist of acatalytic proteins. These are named carbonic anhydrase related proteins (CARPs), and their inactivity is due to absence of one or more Zn-binding histidine residues. In this study, we analyzed and evaluated the distribution of genes encoding CARPs in different organisms using bioinformatic methods, and studied their expression in mouse tissues using immunohistochemistry and real-time quantitative PCR.

**Results:**

We collected 84 sequences, of which 22 came from novel or improved gene models which we created from genome data. The distribution of CARP VIII covers vertebrates and deuterostomes, and CARP X appears to be universal in the animal kingdom. *CA10*-like genes have had a separate history of duplications in the tetrapod and fish lineages. Our phylogenetic analysis showed that duplication of *CA10 *into *CA11 *has occurred only in tetrapods (found in mammals, frogs, and lizards), whereas an independent duplication of *CA10 *was found in fishes. We suggest the name *CA10b *for the second fish isoform. Immunohistochemical analysis showed a high expression level of CARP VIII in the mouse cerebellum, cerebrum, and also moderate expression in the lung, liver, salivary gland, and stomach. These results also demonstrated low expression in the colon, kidney, and Langerhans islets. CARP X was moderately expressed in the cerebral capillaries and the lung and very weakly in the stomach and heart. Positive signals for CARP XI were observed in the cerebellum, cerebrum, liver, stomach, small intestine, colon, kidney, and testis. In addition, the results of real-time quantitative PCR confirmed a wide distribution for the *Car8 *and *Car11 *mRNAs, whereas the expression of the *Car10 *mRNA was restricted to the frontal cortex, parietal cortex, cerebellum, midbrain, and eye.

**Conclusions:**

CARP sequences have been strongly conserved between different species, and all three CARPs show high expression in the mouse brain and CARP VIII is also expressed in several other tissues. These findings suggest an important functional role for these proteins in mammals.

## Background

Carbonic anhydrases (CAs), EC 4.2.1.1, are metal-containing enzymes that occur abundantly in nature and are found in almost all organisms that have been studied [1]. CAs are fundamental to many biological processes, such as photosynthesis, respiration, renal tubular acidification, and bone resorption [2-5]. These enzymes are encoded by five distinct and evolutionarily unrelated gene families known as α, β, γ, δ, and ζ CAs [6]. Interestingly, there is no sequence similarity between these different families. Thus, the CA families are excellent examples of the convergent evolution of catalytic function. The animal kingdom CAs belong to a single gene family known as α-CAs that contain zinc as a metal ion in the active site. The main chemical reaction catalyzed by CAs involves the reversible hydration of CO_2 _(CO_2_+H_2_O (HCO_3_^-^+H^+^).

In mammals, α-CAs are characterized by 16 different isoforms, 13 of which (CA I, II, III, IV, VA, VB, VI, VII, IX, XII, XIII, XIV, and XV) are enzymatically active, whereas the other 3, namely, the CA-related proteins (CARPs) VIII, X, and XI, appear to lack CA activity because of substitutions to 1 or more of the 3 functionally important histidine residues [3,7,8]. In addition, the receptor-type protein tyrosine phosphatases β and γ (RPTP β, RPTP γ) also contain 'CA-like' domains [3,9]. The 13 active CA isozymes differ in their subcellular localizations such that CAs I, II, III, VII, and XIII are all cytosolic enzymes, CAs IV, IX, XII, XIV, and XV are all membrane-associated enzymes, CAs VA and VB are mitochondrial, and CA VI is a secreted protein.

Previous studies on the distribution of CARPs using either western blot analysis or RT-PCR methods have shown a wide expression profile in all parts of the brain in both humans and mice [10-13]. Notably, immunohistochemical studies on CARPs have been mainly focused on brain tissues. The results have shown predominant expression of CARP VIII in the mouse and human cerebellum, especially in the Purkinje cells. Studies on CARP X and XI have revealed a lower level of expression in the cerebellum [14-16]. Previous investigations using reverse transcription polymerase chain reaction (RT-PCR), northern blot analysis, western blot assays or dot blots have reported restricted expression of all CARPs in some mouse and human tissues including the brain [13,17]. The presence of CARPs in the human and mouse brain has suggested important roles for these proteins in the brain development and/or neural functions [12,16]. Interestingly, CARP VIII and XI may also be involved in cancer development in the gastrointestinal tract and lungs [13,18-21].

The pivotal physiological function of CARP VIII became clearly evident in recently published reports on CARP VIII mutations in both human and mouse. A study of waddle mice characterized by a spontaneous mutation in the *Car8 *gene showed ataxia and a distinctive lifelong gait disorder [22]. Another recent study described mild mental retardation, quadrupedal gait and ataxia in members of an Iraqi family who each possessed a defect in the *CA8 *gene [23]. These studies clearly indicated that CARP VIII plays an important role in motor coordination.

The three-dimensional structure of CARP VIII has been recently solved [24], as the only protein in the CARP subfamily. The structural basis of catalytic inactivity is confirmed in this study, but currently there is no interpretation to correlate the structure to any function.

The CARP sequences are well conserved throughout all vertebrates, suggesting that CARPs may play biologically important roles in higher organisms. The number of members in the CARP families has increased with the completion of vertebrate genome sequencing projects, but many sequences have been annotated in incomplete or even partly incorrect forms. To date there are no systematic studies on the distribution and phylogenetic analysis of CARP genes across species nor does there exist any parallel study on the distribution of all three CARP proteins and their mRNA levels in mouse tissues. Therefore, we first used bioinformatic methods to identify and evaluate the distribution of CARP genes across different species. We subsequently performed sequence and phylogenetic analysis of CARPs VIII, X, and XI, focusing on vertebrates, and studied the distribution of the three CARP mRNAs using real-time quantitative PCR (qPCR) and the corresponding proteins using immunohistochemistry in different mouse tissues.

## Results

### Bioinformatic survey and comparison of CARP sequences

**Figure 1:**
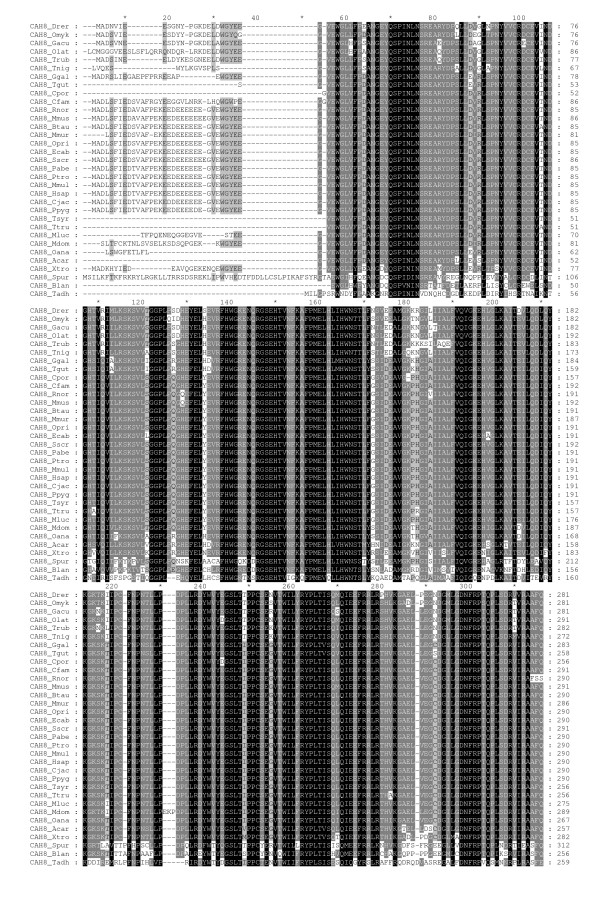
**Multiple sequence alignment of CARP VIII sequences**. Comparison of 33 CARP VIII sequences by multiple sequence alignment. Short names (first letter of the genus and first three letters of the species) are provided on the left side and residue numbers are provided on the right side of the figure. Details of the sequences and full species names are provided in Table 1. The sequences of CAH8_Rnor and CAH8_Spur are trimmed in the end by 21 and 89 residues, respectively.

**Figure 2:**
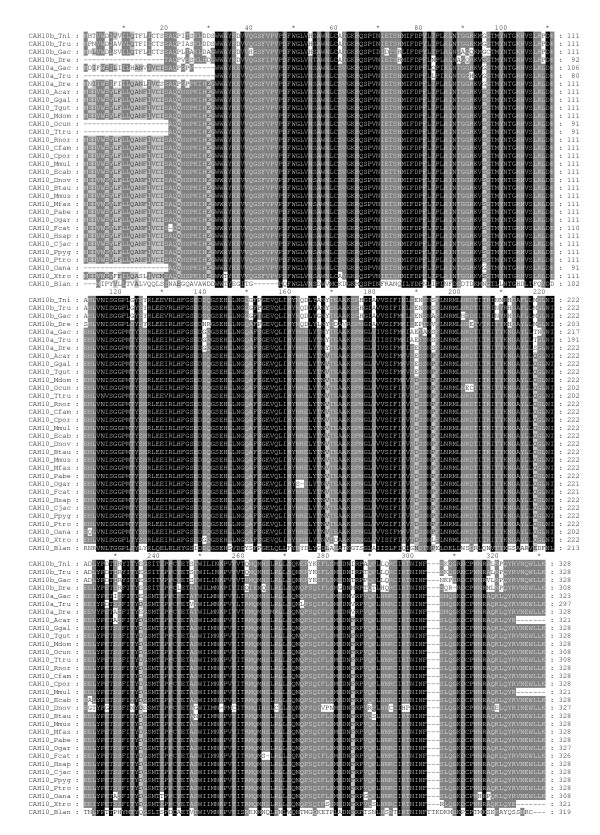
**Multiple sequence alignment of CARP X sequences**. Comparison of 32 CARP X sequences by multiple sequence alignment. Short names (first letter of the genus and first three letters of the species) are provided on the left side and residue numbers are provided on the right. Details of the sequences and full species names are provided in Table 1.

**Figure 3:**
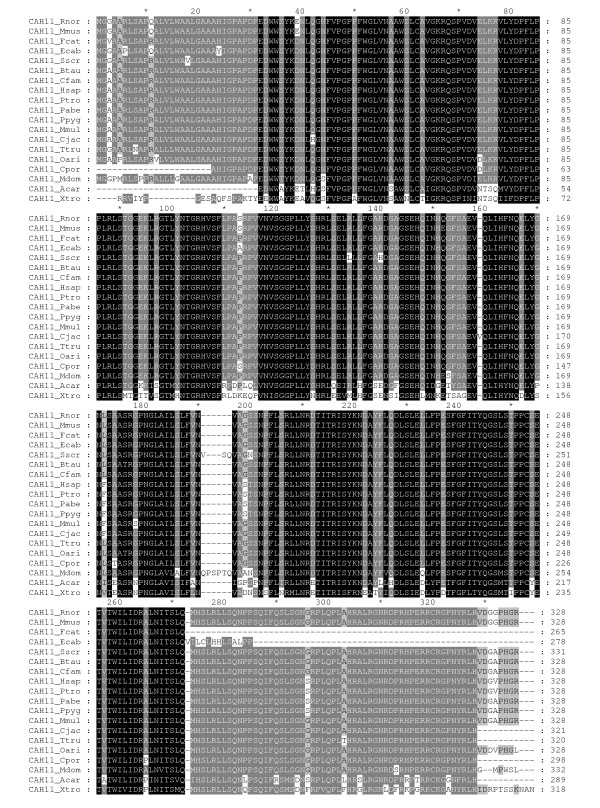
**Multiple sequence alignment of CARP XI sequences**. Comparison of 19 CARP XI sequences by multiple sequence alignment. Short names (first letter of the genus and first three letters of the species) are provided on the left side and residue numbers are provided on the right. Details of the sequences and full species names are provided in Table 1.

Detailed information and database sources from which sequences were obtained are shown in Table 1. In total, 84 full-length sequences were obtained from 38 organisms (3 invertebrates and 35 vertebrates). Thirty-three gene sequences encoding for CARP VIII were identified, of which 3 sequences were from deuterostome invertebrates, namely, *Branchiostoma floridae*, *Trichoplax adhaerens*, and *Strongylocentrotus purpuratus*, and 30 were from vertebrates. We identified 31 sequences of CARP X and 19 sequences of CARP XI in these vertebrates, which included a single CARP X gene from a chordate (*Branchiostoma floridae*). CARP X-like sequences in invertebrates were discovered in both protostomes and deuterostomes. A detailed analysis of invertebrate CARP X homologs will be reported elsewhere. Multiple sequence alignments (MSAs) of the CARP VIII, X, and XI protein sequences are presented in Figures 1, 2, and 3, respectively.

**Table 1:** Details of the carbonic anhydrase-related protein (CARP) sequences used in this study

Group	Organism	Scientific Name	CARPs	Acc. No./ID	Length (aa)	Description^1^
	Cow	*Bos taurus*	VIII	NP_001077159	290	NCBI_RefSeq
			X	A0JN41	328	Uniprot
			XI	NP_783648.1	328	NCBI_RefSeq
	Marmoset	*Callithrix jacchus*	VIII	-	290	UCSC_BLAT
			X	-	328	UCSC_BLAT
			XI	A6MLD1	321	Uniprot _UCSC_BLAT
	Dog	*Canis familiaris*	VIII	XP_544094.2	290	NCBI_RefSeq
			X	XP_866307.1	328	Ensembl_UCSC_BLAT
			XI	XP_852031.1	328	NCBI_RefSeq
	Guinea Pig	*Cavia porcellus*	VIII	ENSCPOP00000017966	256	Ensembl
			X	ENSCPOP00000013970	328	Ensembl
			XI	ENSCPOP00000011461	332	Ensembl
	Armadillo	*Dasypus novemcinctus*	X	ENSDNOP00000008341	327	Ensembl
	Horse	*Equus caballus*	VIII	XP_001496523.1	290	NCBI_RefSeq
			X	XP_001503286.2	328	NCBI_RefSeq
			XI	ENSECAP00000015876	278	Ensembl
	Cat	Felis catus	X	ENSFCAP00000002102	326	Ensembl_UCSC_BLAT
			XI	ENSFCAP00000006798	265	Ensembl
	Human	*Homo sapiens*	VIII	P35219	290	Uniprot
			X	Q9NS85	328	Uniprot
			XI	O75493	328	Uniprot
	Mouse	*Mus musculus*	VIII	P28651	291	Uniprot
			X	P61215	328	Uniprot
			XI	O70354	328	Uniprot
	Macaque	*Macaca mulatta*	VIII	XP_001088977.1	290	NCBI_RefSeq
			X	XP_001101492.1	334	NCBI_RefSeq
			XI	XP_001113730.1	328	RefSeq_UCSC_BLAT
	Rhesus Monkey	*Macaca fascicularis*	X	Q9N085	328	Uniprot
	Mouse Lemur	*Microcebus murinus*	VIII	ENSMICP00000001089	286	Ensembl
**Mammals**	Opossum	*Monodelphis domestica*	VIII	ENSMODP00000010658	289	Ensembl_UCSC_BLAT
			X	ENSMODP00000015705	328	Ensembl_UCSC_BLAT
			XI	ENSMODP00000017625	332	Ensembl
	Microbat	*Myotis lucifugus*	VIII	ENSMLUP00000008230	275	Ensembl
	Pika	*Ochotonidae princeps*	VIII	ENSOPRP00000014387	290	Ensembl
	Platypus	*Ornithorhynchus anatinus*	VIII	ENSOANP00000008002	267	Ensembl
			X	ENSOANP00000003611	308	Ensembl_UCSC_BLAT
	Rabbit	*Oryctolagus cuniculus*	X	ENSOCUP00000002025	333	Ensembl
	Bushbaby	*Otolemur garnettii*	X	ENSOGAP00000004458	327	Ensembl
	Chimpanzee	*Pan troglodytes*	VIII	XP_519778.2	290	NCBI_RefSeq
			X	XP_001171455.1	328	NCBI_RefSeq
			XI	XP_001171520.1	328	RefSeq_UCSC_BLAT
	Sumatran Orangutan	*Pongo abelii*	VIII	-	290	UCSC_BLAT
			X	Q5R4U0	328	Uniprot
			XI	NP_001128968.1	328	NCBI_RefSeq
	Orangutan	*Pongo pygmaeus*	VIII	ENSPPYP00000020875	290	Ensembl
			X	ENSPPYP00000009287	328	Ensembl
			XI	ENSPPYP00000011415	328	Ensembl
	Rat	*Rattus norvegicus*	VIII	Q5PPN4	290	Uniprot
			X	EDM05681	319	NCBI_RefSeq
			XI	NP_783639	328	NCBI_RefSeq
	Pig	*Sus scrofa*	VIII	XP_001926916	291	NCBI_RefSeq_EST
			XI	Q866X6	331	Uniprot
	Tarsier	*Tarsius syrichta*	VIII	ENSTSYP00000009029	256	Ensembl
	Dolphin	*Tursiops truncatus*	VIII	ENSTTRP00000015250	256	Ensembl
			X	ENSTTRP00000001587	308	Ensembl
			XI	ENSTTRP00000007758	320	Ensembl
	Sheep	*Ovis aries*	XI	Q95203	328	Uniprot

	Anole Lizard	*Anolis carolinensis*	VIII	-	257	UCSC_BLAT
			X	-	321	UCSC_BLAT
			XI	ENSACAP00000015571	289	Ensembl
	Chicken	*Gallus gallus*	VIII	ENSGALP00000024873	283	Ensembl
			X	XP_415644.1	328	NCBI_RefSeq
	Zebra Finch	*Taeniopygia guttata*	VIII	ENSTGUP00000011522	258	Ensembl
			X	ENSTGUP00000009733	328	Ensembl
	Frog	Xenopus tropicalis	VIII	Q5M8Z5	282	Uniprot
			X	ENSXETP00000002773	321	Ensembl_UCSC_BLAT
			XI	-	303	UCSC_BLAT
	Zebrafish	Danio rerio	VIII	NP_001017571.1	281	NCBI_RefSeq
Other			Xa	NP_001032198.1	328	NCBI_RefSeq
vertebrates			Xb	XP_696967.2	308	NCBI_RefSeq
	Stickleback	Gasterosteus aculeatus	VIII	ENSGACP00000019684	281	Ensembl
			Xa	ENSGACP00000020102	323	Ensembl_UCSC_BLAT
			Xb	ENSGACP00000000619	328	Ensembl
	Rainbow trout	Oncorhynchus mykiss	VIII	Q5I2J1	281	Uniprot
	Medaka	Oryzias latipes	VIII	ENSORLP00000016815	281	Ensembl
	Fugu	Takifugu rubripes	VIII	ENSTRUP00000009702	282	Ensembl
			Xa	ENSTRUP00000007839	297	Ensembl_UCSC_BLAT
			Xb	ENSTRUP00000032938	328	Ensembl_UCSC_BLAT
	Pufferfish	Tetraodon nigroviridis	VIII	ENSTNIP00000005959	272	Ensembl
			Xa	ENSTNIP00000017020	328	Ensembl_UCSC_BLAT

	Lancelet	Branchiostoma lancelet	VIII	C3XTT7	256	Uniprot_UCSC_BLAT
			X	-	319	UCSC_BLAT
Invertebrates	Trichoplax	Trichoplax adhaerens	VIII	XP_002111595.1	348	NCBI_RefSeq
	Sea urchin	Strongylocentrotus purpuratus	VIII	XP_795365.2	312	NCBI_RefSeq

^1 ^The sources of the best protein sequences. NCBI, RefSeq, UniProt and Ensembl refer to the respective databases, and UCSC_BLAT refers to discovery of novel genes/exons using the UCSC genome browser and subsequent gene model building, as detailed in Materials and Methods. EST means that a further EST sequence was used to extend an incomplete genome sequence.

Among vertebrate sequences, the protein sequence identities ranged from 67% to 100% for CARP VIII, from 70% to 100% for CARP X and from 70% to 100% for CARP XI. The 100% values were observed between primate sequences. However, even between humans and mice, identities of CARP orthologs were 98%, 100%, and 96% for CARP VIII, X, and XI, respectively. When the invertebrate CARP VIII and CARP X sequences used in this study were compared to their vertebrate orthologs, the sequence identities were only 40% to 45%. For comparison, in case of enzymatically active CAs, protein sequence identity between human and mouse are 94%, 93%, 92%, and 91% for CAs VII, Vb, III, and XIII, respectively, and 50% to 83% for other pairs of isozymes. Thus, sequence conservation was found to be higher in all CARPs than in any of the active CAs.

### Phylogenetic analysis

**Figure 4:**
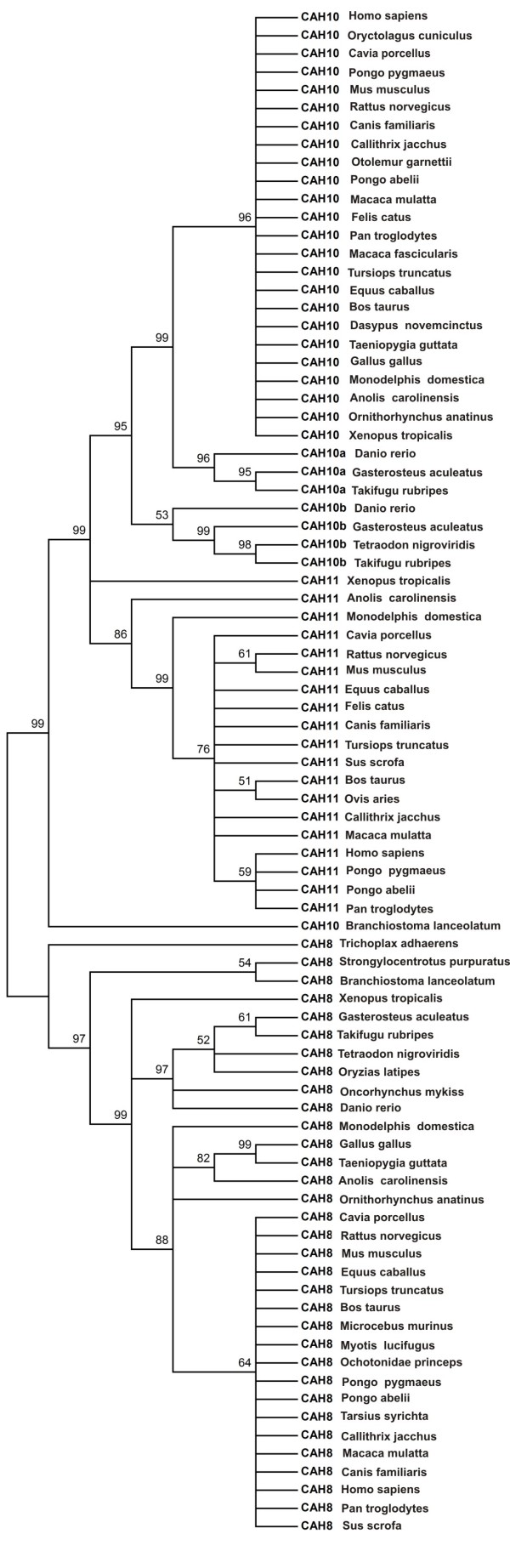
**Evolutionary relationships of CARP sequences**. The phylogenetic tree of 84 CARP sequences used in the study was inferred using the Neighbor-Joining method. The percentage of replicate trees in which the associated sequences clustered together in the bootstrap test is shown above the branches.

The phylogenetic tree of the CARP protein sequences is shown in Figure 4. The full MSA from which the tree was calculated is provided as Additional file 1. The tree shows two distinct sequence pools, including one pool for CARP VIII and the other pool for CARPs X and XI. The CARP VIII pool subscribes to the expected animal taxonomy, except for mammals, which are not resolved due to sequence identities near 100%. The second sequence pool is comprised of the CARP X and CARP XI sequences. CARP X forms three subgroups, including the large subgroup consisting of sequences from mammals, frogs, and lizards, and two smaller groups containing sequences from fishes (Figure 4). The CARP XI group contains a major branch, consisting of mammalian sequences, while the sequences from lizard and frog form an outgroup. The tree indicates that there has been an independent duplication of the ancestral vertebrate *CA10 *gene in the fish lineage, whereas we conclude that the *CA11 *gene has emerged from another gene duplication after the separation of the fish and tetrapod lineages.

### Quantitative analysis of *Car 8, Car10, Car11 *mRNA expression in mouse tissues

**Figure 5:**
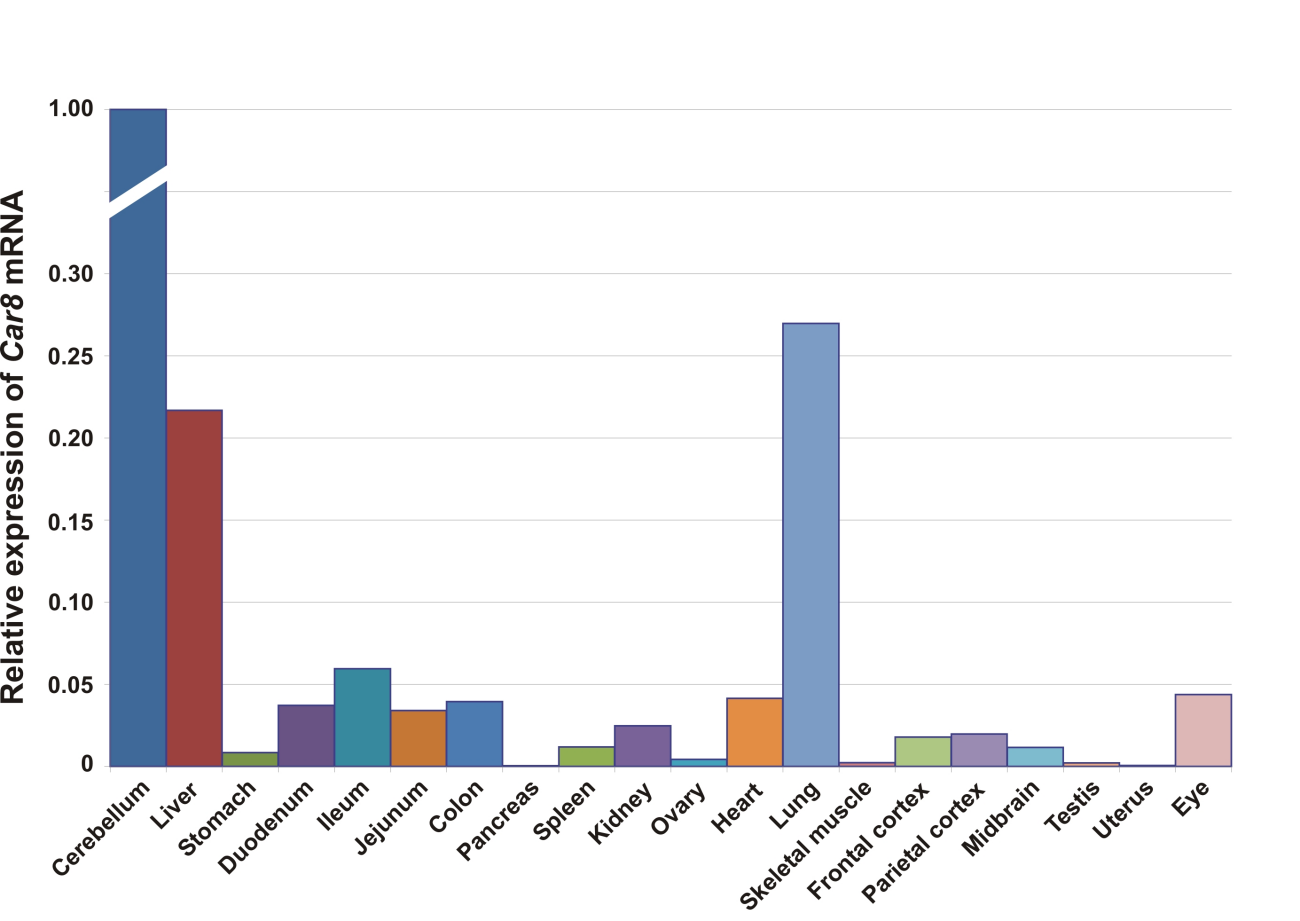
**Expression pattern of *Car8 *mRNA in different murine tissues**.

We studied the expression of all three CARP mRNAs in 20 different mouse tissues. The expression patterns of each mRNA are shown in Figures 5 through 7. As predicted based on previous studies [15], *Car8 *mRNA expression was found to be highest in the cerebellum, and high levels were also detected in the liver and the lung (Figure 5). Low expression was observed in the stomach, duodenum, ileum, jejunum, colon, spleen, kidney, heart, frontal cortex, parietal cortex, midbrain, and eye, while extremely low expression was observed in the ovary, skeletal muscle, and testis.

**Figure 6:**
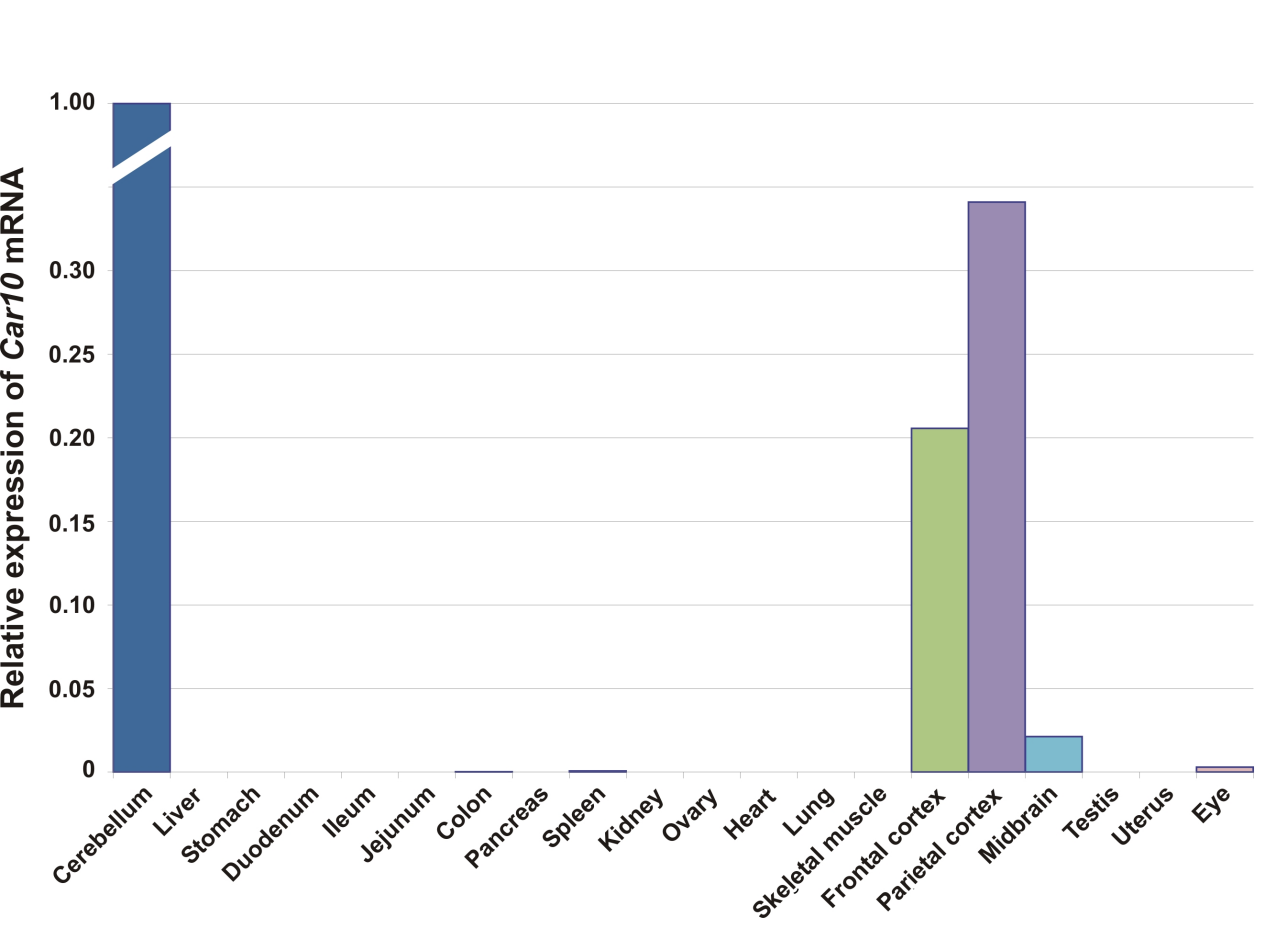
**Expression profile of *Car10 *mRNA in murine tissues**.

**Figure 7:**
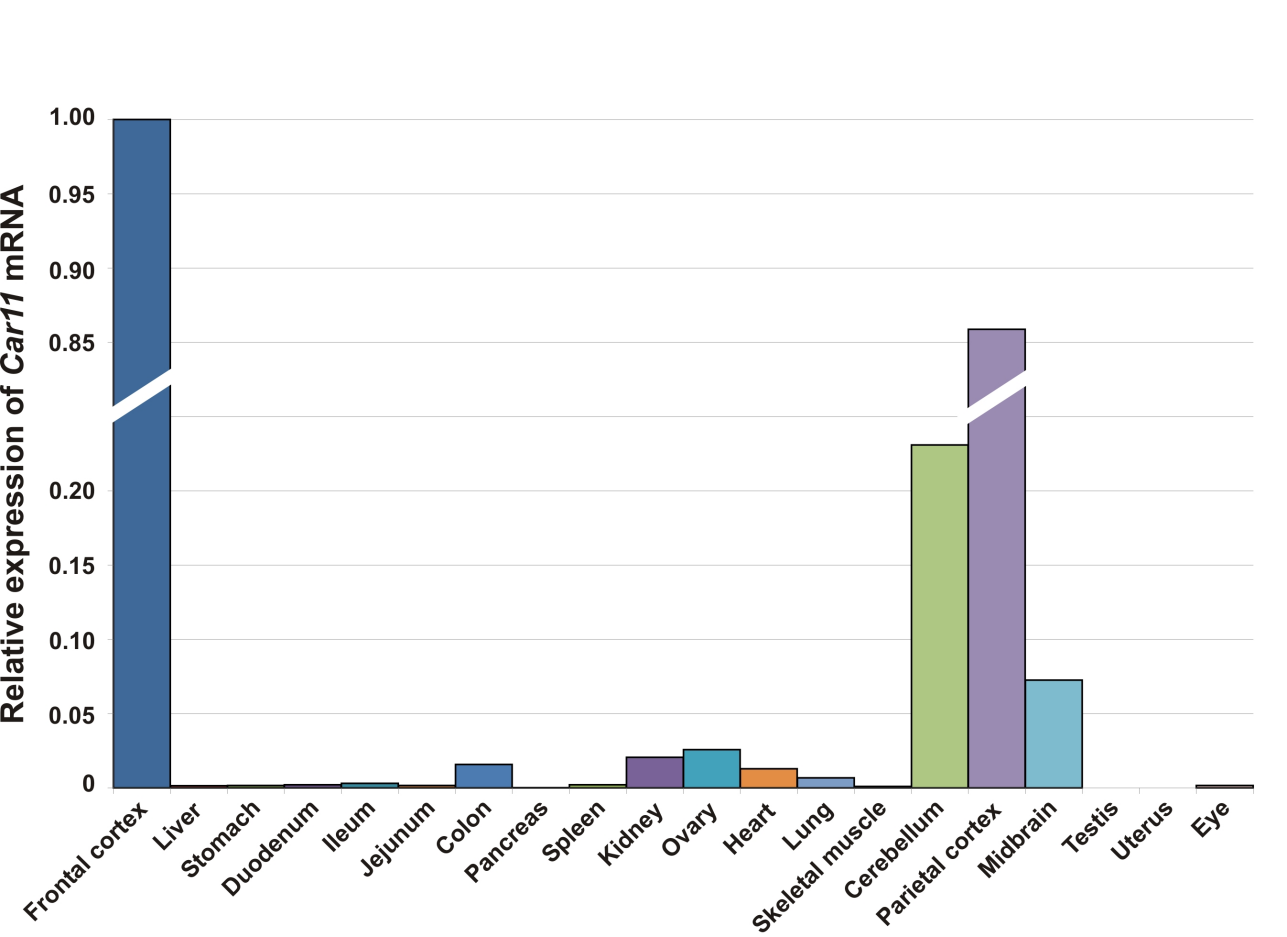
**Distribution of *Car11 *mRNA in murine tissues**.

The expression profile for *Car10 *mRNA is presented in Figure 6. The *Car10 *mRNA levels were high in the cerebellum, frontal cortex, and parietal cortex, low in the midbrain, and extremely low in the eye. The distribution of *Car11 *mRNA expression was broad, and the highest signals were observed in the cerebellum and cerebral cortex (Figure 7) while the midbrain showed moderate levels of expression. In addition to the brain, low expression was detected in the colon, kidney, ovary, heart, and lung. Moreover, barely detectable expression was observed in the liver, stomach, duodenum, ileum, jejunum, spleen, and eye.

### Distribution of CARP VIII, X, and XI proteins in mouse tissues

**Figure 8:**
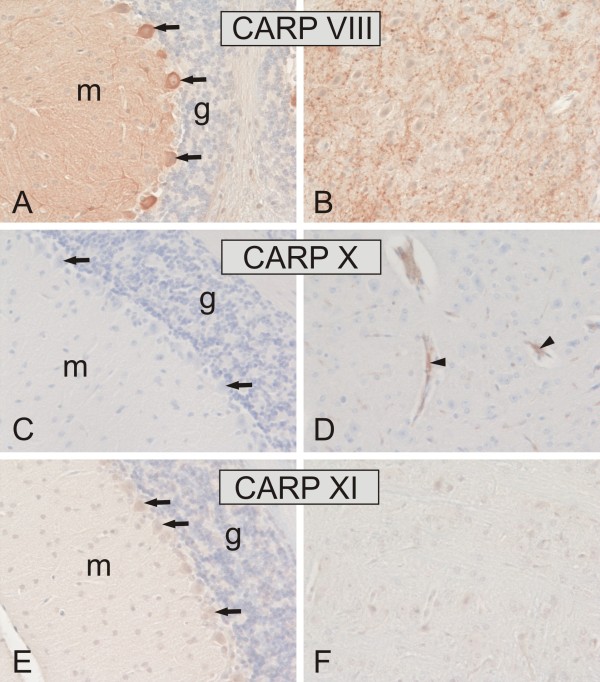
**Immunohistochemical staining of CARP VIII, CARP X, and CARP XI proteins in mouse cerebellum (A, C, E) and cerebrum (B, D, F)**. Arrows indicate the location of the Purkinje cells, which are strongly positive for CARP VIII and moderately positive for CARP XI. The molecular layer (m) of the cerebellum is also intensely labeled with CARP VIII, whereas the granular cell layer is negative (g). Panel B shows strong punctuate immunostaining for CARP VIII in the cerebrum. The arrowheads in panel D indicate CARP X-positive microcapillaries in the cerebrum. Immunostaining reactions for CARP XI remained very weak in the cerebrum (F). Original magnifications are at × 20.

**Figure 9:**
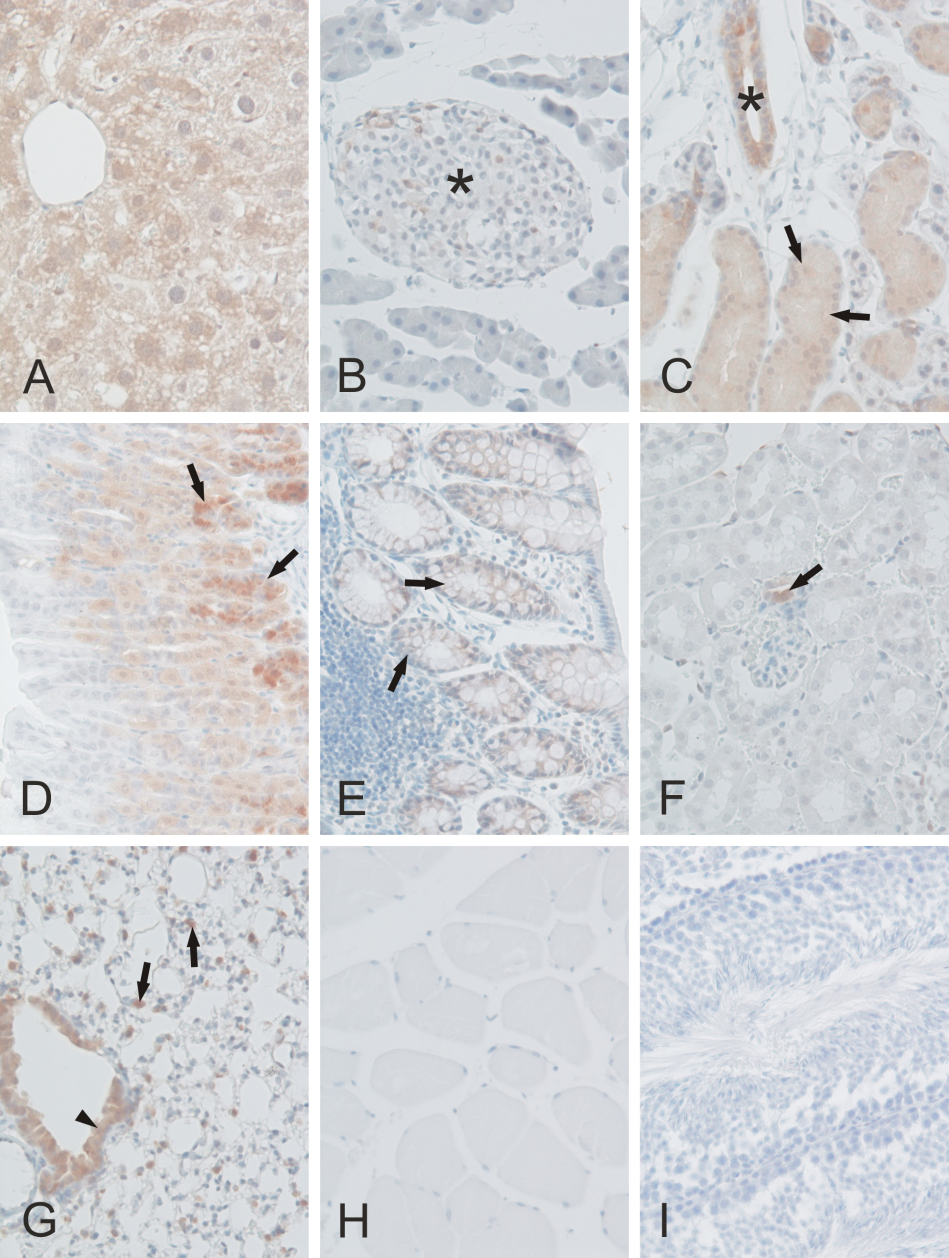
**Immunohistochemical staining of CARP VIII proteins in mouse tissues**. CARP VIII was observed moderately in the liver hepatocytes (A), ducts (*) and acini (arrows) of the submandibular gland (C), gastric glands (arrows in D), respiratory epithelium (arrowhead), and rounded alveolar cells (arrows) of the lung (G). Extremely low expression was observed in the pancreatic Langerhans islets (*in B), colonic glands (arrows in E), and occasionally in the tubule cells of the kidney (arrow in F indicates positive macula densa cells). No staining was present in the skeletal muscle (H) and testis (I). Original magnifications are at × 20.

We studied the expression of CARP VIII, X, and XI proteins in mouse tissues using immunohistochemistry as shown in Figures 8 through 11 and in Table 2. CARP VIII was expressed in most of the tissues analyzed, indicating a wider distribution profile compared to CARP X and CARP XI expression. Strong expression for CARP VIII was observed in the cerebellum and cerebrum while weaker expression was observed in several other tissues including the liver, pancreatic Langerhans islets, submandibular gland, stomach, colon, kidney, and lung (Figures 8 and 9). In the cerebellum, the highest staining intensity was present in the Purkinje cells and a slightly lower staining intensity was associated with the molecular layer. The cerebrum showed an intense and punctate staining pattern, indicating the strongest expression in the axons and dendrites. In the kidney, very weak positive staining was observed in a few epithelial cells of the renal tubules. The liver showed moderate immunostaining in the hepatocytes. In the lung, staining was observed in both the respiratory epithelium and the rounded alveolar cells, most likely representing the type II pneumocytes. The submandibular gland showed strong immunoreactions in both the acinar and ductal epithelial cells. The gastric and colonic glands were also positively stained for CARP VIII.

**Table 2:** Expression profile of CARP VIII, X, and XI in mouse tissues using immunohistochemistry

Tissue	CARP VIII	CARP X	CARP XI
Cerebellum	+++	---	++-
Cerebrum	+++	++-	+--
Liver	++-	---	+--
Pancreas	+--	---	---
Submandibular gland	++-	---	---
Stomach	++-	+--	+--
Colon	+--	---	+--
Kidney	+--	++-	+--
Lung	++-	---	---
Skeletal muscle	---	---	--
Testis	---	NA	+-
Ovary	NA	--	NA
Heart	NA	+--	NA
Epididymis	NA	---	---
Small intestine	---	---	+--

+++ Strong, ++Moderate, +-Weak, ---No signal, and NA-Not analyzed

**Figure 10:**
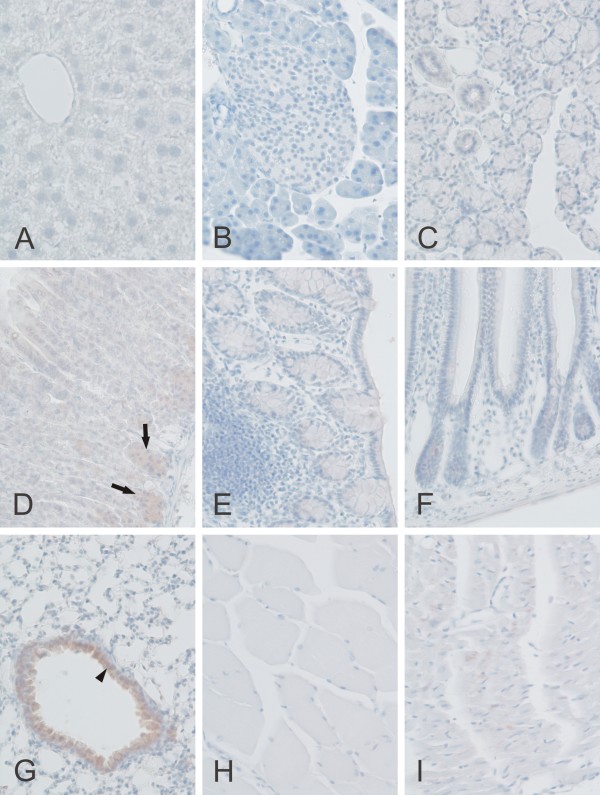
**Immunohistochemical staining of CARP X protein in mouse tissues**. Moderate CARP X expression was observed in the respiratory epithelium (arrowhead) of the lung (G). Moderate expression was also present in the gastric glands (arrows in panel D). The heart muscle cells occasionally showed extremely weak signals (I). The other tissues including the liver (A), pancreas (B), submandibular gland (C), colon (E), small intestine (F), skeletal muscle (H) remained negative. Original magnifications are at × 20.

A significant amount of CARP X expression was observed only in the lung where the staining was localized to the respiratory epithelium. In addition, positive signals were occasionally detected in the cerebral capillaries and the stomach (Figures 8 and 10 and Table 2). Barely detectable staining was occasionally observed in the heart muscle cells.

**Figure 11:**
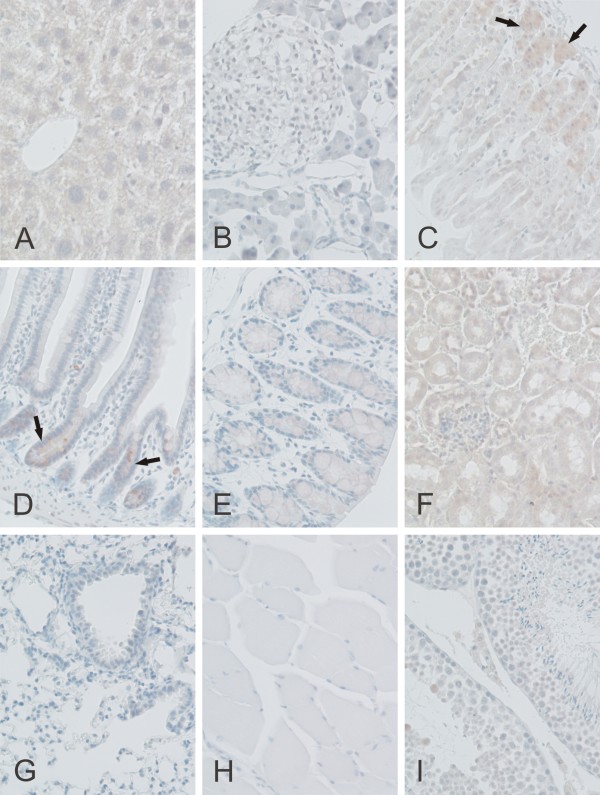
**Immunohistochemical staining of CARP XI protein in mouse tissues**. Weak immunoreactions for CARP XI were observed in the crypts of Lieberkuhn (arrows in D), gastric glands (arrows in C), and renal tubule cells (F). Extremely weak signals can also be observed in the liver (A), colon (E), and testis (I). The pancreas (B), lung (G), and skeletal muscle (H) were all negative. Original magnifications are at × 20.

CARP XI showed a broader expression profile than CARP X. Its overall distribution was fairly similar to that for CARP VIII, although the staining intensity was clearly less intense. Positive signal for CARP XI was observed in the cerebellum, cerebrum, liver, stomach, small intestine, colon, kidney, and testis (Figures 8 and 11 and Table 2). In the cerebellum, the most prominent signal was located in the Purkinje cells.

## Discussion

Previous bioinformatic investigations have described individual CARP sequences only for human, mouse, and other mammals [10,11,25,26]. Using a variety of bioinformatic tools we identified 84 full-length sequences from genome and sequence databases, including 22 coming from novel or improved gene models and combining mRNA data to genome data. Of these 22 sequences, 8 are novel and previously unannotated, and 14 comprise extended and/or partially corrected sequences. Sequences encoding CARP VIII and CARP X were identified in all available vertebrate genomes, even if many sequences were present only as gene fragments. In contrast, CARP XI was found only in mammals, frogs (*X. tropicalis*), and lizards (*A. carolinensis*). Our results indicate that the *CA11 *gene emerged through a process of gene duplication from *CA10 *after the divergence of the fish and tetrapod lineages. In addition, we failed to identify any CARP XI sequences from birds, but since there are only two genomes available, the *CA11 *gene may have been either missed as a consequence of the incomplete genome coverage or alternatively the gene may have been lost in the bird lineage.

The fish CARP X-like sequences form two distinct subgroups, both of which are more closely related to CARP X than CARP XI. This indicates that these sequences have arisen by gene duplication from the *CA10 *gene in the fish lineage. We suggest names *CA10a *and *CA10b *for these genes and CARP Xa and CARP Xb for the proteins to distinguish them from *CA11*/CARP XI, which is specific to tetrapods.

Sequences found in three deuterostomes (*B. floridae*, *T. adhaerens*, and *S. purpuratus*) that were similar to CARP VIII, with sequence similarities between 40% and 45% when compared to vertebrate CARP VIII sequences, were novel and unexpected discoveries. No CARP VIII orthologs were likewise discovered in protostomes. According to these findings, the origin of CARP VIII would have occurred sometime after the separation of the Protostomia and Deuterostomia lineages. In contrast, CARP X-like sequences are widely found in invertebrates, including all available insect and nematode genomes. Further analysis of the invertebrate CARP X homologs will be presented elsewhere.

Earlier studies reported the expression of human or mouse CARP mRNAs and their corresponding proteins using immunohistochemistry, western blot analysis, northern blot assays, dot blots, and RT-PCR mostly in the brain and in a few cases in other tissues, often with slightly conflicting results [12,13,16,22,27-30]. However, a comprehensive distribution of all CARPs and their corresponding mRNAs has yet to be completely elucidated. In fact, the present study revealed for the first time the distribution of all CARP proteins and their mRNAs in a wide variety of adult mouse tissues using immunohistochemistry and real-time qPCR.

The expression of *Car8 *mRNA has been previously studied in some other tissues besides from the brain using RT-PCR and dot blot analysis. Multiple transcripts were reported to be expressed in the brain, lung, and liver, and a single transcript was found in the heart, skeletal muscle, and kidney, though to a lesser extent [11]. In the present study, we observed high expression of *Car8 *mRNA in the cerebellum, liver, and lung. Low expression of *Car8 *mRNA was observed in all other tissues except for the pancreas and uterus. In brief, the expression pattern we observed by RT-qPCR was similar to the earlier results [11]. The immunohistochemical findings show high levels of CARP VIII expression in the cerebellum and cerebrum. In previous studies, immunohistochemical staining, northern blot studies, and RT-PCR analyses have shown abundant expression of CARP VIII in the human and mouse brain, and immunohistochemical studies have further defined its expression especially in the cerebellar Purkinje cells [12,13,16,22,27-30], all of which are in agreement with the results of our study. Lower CARP VIII expression has been reported in other murine tissues including the lung, liver, and stomach using western blot analysis [30]. Our study confirmed the expression using immunohistochemical staining in these organs. High level expression of *Car8 *mRNA and CARP VIII protein in the brain and in a wide variety of other tissues suggests important roles for this protein in normal physiology. Indeed the role of CARP VIII is evident from the recently published reports showing ataxia and gait disorders in both mice and humans due to mutations in the *CA8 *gene [22,23].

RT-PCR analyses have previously shown the expression of *Car10 *mRNA in the human brain, testis, salivary glands, and kidney, while lower expression levels were reported in the pancreas, liver, and testis [11]. Using northern blot analysis, the expression was observed in the kidney and the brain [11]. Incidentally, there have been only a few previous reports in the literature regarding the expression of the CARP X protein in the human and mouse brain. The expression was shown to be weak in the cerebellar Purkinje cells [14,16]. Herein we report strong positive signal intensities only in the cerebellum followed by the parietal cortex and the frontal cortex, low expression in the midbrain, and extremely low expression in the eye. Using immunohistochemical studies, we observed clearly localized signal only in the respiratory epithelium of the lung, and weak signals in the stomach and cerebral capillaries. Our real-time qPCR results do not agree with previous results on human tissues (except for the brain) nor do they agree with the present immunohistochemical findings. The discrepancies between immununohistochemistry and RT-qPCR may be due to (i) a low amount of mRNA that is translated into protein; (ii) rapid degradation of the protein; (iii) the low signal from immunochemical staining, which could be due to the loss of antigenicity in some tissues during the processing and storage of slides; (iv) differences in the species or strains used. Further immunohistochemical studies using new antibodies along with analysis of mRNA transcripts will be important for understanding the discrepancies observed.

In a previous paper, Okamoto et al. [11] reported that the *Car10 *sequence contains seven CCG repeats in the 5'-untranslated region followed by two CCG repeats located 16 bp downstream from the aforementioned repeats. These repeats have been associated with various neurological disorders [31]. The presence of the CCG repeats in the *Car10 *gene makes it a potential candidate gene that might contribute to the development of neurodegenerative disorders. Therefore, it will be of interest to explore the expansion mutations of *Car10 *gene in patients with neurological symptoms [8].

Our study revealed widespread expression of CARP XI in most of the tissues studied by using both immunohistochemistry and real-time qPCR. Compared to CARP VIII, the intensity of CARP XI immunostaining was clearly weaker. In a previous study, immunochemical staining of the human brain indicated that the signal for CARP XI was lower compared to CARP VIII but higher than CARP X [16]. Thus, our findings were in agreement with these aforementioned results. The present real-time qPCR analysis, surprisingly, showed very high expression of the *Car11 *mRNA in all of the brain segments analyzed, especially in the frontal cortex followed by the parietal cortex, cerebellum, and midbrain. These results were in agreement with an earlier report showing *Car11 *mRNA expression in all parts of the human brain [10]. The same study demonstrated *Car11 *mRNA expression in the kidney, liver, and salivary glands, and low expression levels in the lung, skeletal muscle, kidney, pancreas, and liver [10]. In our study, low levels of *Car11 *mRNA were observed in the colon, kidney, ovary, heart, and lung. The presence of CARP XI in several regions of the brain suggests an important, yet undefined role for CARP XI in the central nervous system.

## Conclusions

The present investigation describes a comprehensive bioinformatic study of CARP gene sequences and also elucidates the distribution of three CARPs in mouse tissues using real-time qPCR and immunohistochemistry. We have observed a very high conservation of all the three CARP sequences across the species. In the cases of CARP VIII and CARP X, we found unusually high similarity between vertebrate and invertebrate sequences. Based on our results, the duplication history of the *CA10 *gene has followed different paths in the fish and tetrapod lineages. Our results contribute to a deeper understanding of CARP evolution across species.

The expression of CARP VIII was found to be widespread in the tissues analyzed, and the highest mRNA signals were detected in the cerebellum, lung, and liver. Both CARP X and XI showed the strongest mRNA expression in the nervous tissues. The distribution patterns suggest that CARPs may contribute to the development of the nervous system, motor coordination functions, and yet unknown physiological roles in other tissues. It will be of interest to determine the specific function of all CARPs by producing and analyzing suitable single, double, and triple knockout animal models and also by screening neurological patients for trinucleotide repeats or other mutations in CARP genes.

## Methods

### Sequence retrieval

The available CARP protein sequences were obtained from Ensembl [32], UniProt [33], and RefSeq [34] and further sequences were searched using BLAST from NCBI protein databases [35] and via BLAT searches from complete genomes [36] using human and mouse CARPs as initial query sequences, and zebrafish, lancelet and sea urchin CARPs later as they were discovered and confirmed. Duplicate sequences were rejected, after which the remaining sequences were taken through iterated cycles of multiple sequence alignment [37], evaluation, and revision. For revision, sequences with poorly matching or missing regions were subjected to gene model generation with GeneWise [38] taking the genomic sequences from the UCSC Genome Browser [36]. EST and mRNA sequence data were used to confirm gene models, to bridge gaps or fill ends in the genomic sequences, and to discover and assemble CARPs from less than genome-wide sequenced organisms. Finally, incomplete sequences were rejected, with the exception of marginally shortened ends, which were allowed.

### Multiple sequence alignment

Individual multiple sequence alignments (MSAs) were calculated for CARP VIII, CARP X, and CARP XI using ClustalW [39] and visualized using GeneDoc software [40]. Furthermore, all of the 84 CARP protein sequences were aligned together for the phylogenetic tree.

### Phylogenetic analysis

The phylogenetic tree of all CARP protein sequences was constructed from the MSA of 84 sequences using the MEGA software, version 4.1 [41]. Evolutionary relationships were inferred using the Neighbor-Joining method. A bootstrap test was performed using 1000 replicates and evolutionary distances were computed using the Poisson correction method with the complete deletion option.

### Immunohistochemistry

The tissue specimens from the normal mice were fixed in 4% neutral-buffered formaldehyde at +4°C for 8 to 27 days. The samples were then dehydrated in an alcohol series, treated with xylene, embedded in paraffin wax, and 4 μm sections were cut and placed on Superfrost microscope slides. After removal of the paraffin with xylene, the rehydrated sections were boiled in sodium citrate (0.01 M, pH 6.0) for 20 min and cooled down. The sections were immunohistochemically stained according to the following procedure: (i) incubating the sample in methanol + 3% H_2_O_2 _for 5 min; (ii) rinsing with 1 × Tris-buffered saline (TBS), pH 8.0, containing 0.05% Tween; (iii) blocking with Rodent Block M™(Biocare Medical, Concord, CA) for 30 min; (iv) incubating with primary rabbit anti-human CARP VIII, CARP X, and CARP XI antibodies (Santa Cruz Biotechnology, Inc. Bergheimer Heidelberg, Germany) raised against amino acids 1 to 100 (CARP VIII), 1 to 50 (CARP X) and 279 to 328 (CARP XI) diluted 1:350 in 1% bovine serum albumin (BSA) in phosphate-buffered saline (PBS) for 1 hour at room temperature and rinsing with TBS containing 0.05% Tween. Notably, rabbit IgG was used as a control instead of the primary antibodies; (v) incubation with Rabbit HRP-Polymer + XM Factor™(Biocare Medical, Concord, CA) (2.5 ml HRP-Polymer + 1 to 2 drops XM Factor) for 30 min prior to rinsing with 1 × TBS containing 0.05% Tween; (vi) treatment with 1 × 3,3'-diaminobenzidine tetrahydrochloride (DAB) solution for 5 min prior to rinsing with distilled water; (vii) counterstaining of the slides with Mayer's Hematoxylin for 1 to 3 sec and rinsing under tap water for 10 min. After dehydration, the slides were mounted with Entellan Neu™ (Merck; Darmstadt, Germany), examined and photographed using Nikon Microphot microscope (Nikon Microphot-FXA, Japan). All of the procedures were carried out at room temperature.

### RNA Extraction

Specimens from 20 tissue samples were collected from six normal NMRI mice. The tissue samples used for mRNA isolation were stabilized in RNAlater (Ambion, Austin, TX, USA) immediately after collection, and the total RNA was isolated from 30 mg of tissue samples using the RNeasy Mini kit (Qiagen, Hilden, Germany) by following the manufacturer's instructions. The concentration and purity of RNA was determined using a spectrophotometric method at 260 and 280 nm.

### Quantitative real-time PCR

Reverse transcriptase PCR was performed using 50 μg of total RNA to synthesize the first strand of cDNA using First Strand cDNA Synthesis kits (High-Capacity cDNA Reverse Transcription Kits, Applied Biosystems, Foster City, CA) with random primers and MultiScribe™ Reverse Transcriptase according to the protocol recommended by the manufacturer.

Real-time qPCR primers were designed based on the complete cDNA sequences deposited in the GenBank (RefSeq accession numbers: *Car8 *[NCBI: NM_007592], *Car10 *[NCBI: NM_028296], and *Car11 *[NCBI: NM_009800]). Primer sequences for *Car8 *included the Forward primer (5'-3') cgggattactgggtctatgaagg and the Reverse primer (5'-3') ggctgggtaggtcggaaattgtc, while the primer sequences for *Car10 *included the Forward primer (5'-3') gagagcaagagcccagaactc and the Reverse primer (5'-3') ctcaccagtggcagaaatggc. In addition, the *Car11 *primer sequences were (5'-3') gccggctctgaacaccagatc (for the Forward primer) and (5'-3') gaggaggcgactgaggaatgg (for the Reverse primer).

Real-time qPCR was performed using the SYBR Green PCR Master Mix Kit in an ABI PRISM 7000 Detection System™ according to the manufacturer's instructions (Applied Biosystems). The PCR conditions consisted of an initial denaturation step at 95°C for 10 min followed by 40 cycles at 95°C for 15 sec (denaturation) and 60°C for 1 min (elongation). The data were analyzed using the ABI PRISM 7000 SDS™ software (Applied Biosystems). Every PCR was performed in a total reaction volume of 15 μl containing 2 μl of first strand cDNA (20 ng cDNA), 1 × Power SYBR green PCR Master Mix™ (Applied Biosystems, Foster City, CA 94404, USA), and 0.5 μM of each primer. The final results, expressed as the *N*-fold relative difference (ratio) in gene expression between the studied samples, were calculated according to the Pfaffl's equation with appropriate modification [42].

## Authors' contributions

All authors participated in the design of the study. AA is the corresponding author who performed the experimental work and drafted the first version of the manuscript. AA and MT jointly performed the bioinformatic analyses, under supervision of MT. In addition, AA and SP performed essential microscopic analyses. All authors contributed to the writing of the document and approved the final version of the manuscript.

## Supplementary Material

Additional file 1 **Multiple sequence alignment of CARP VIII, X, and XI sequences**. Multiple sequence alignment of the 84 CARP protein sequences analyzed in the study which was used for the construction of the phylogenetic tree. Click here for file
