# Supplementary material for: Phylogeny and expression of carbonic anhydrase-related proteins
Source: BMC Mol Biol. 2010 Mar 31;11:25. doi: 10.1186/1471-2199-11-25 (PMC2873310; doi:10.1186/1471-2199-11-25)
Supplement: Additional file 1 — Multiple sequence alignment of CARP VIII, X, and XI sequences. Multiple sequence alignment of the 84 CARP protein sequences analyzed in the study which was used for the construction of the phylogenetic tree. [file 1471-2199-11-25-S1.PDF]

```
                            *        20         *        40         *        60         *        80         *
CAH10_Rnor : -------MEIVWEVLFLLQANFIVCISAQQNSPKIHEGWWAYKEVVQGSFVPVPSFWGLVNSAWNLCSVGKRQSPVNIETSHMIFDPFLT :  83
CAH10_Fcat : -------MEIVWEVLFLLQANFIVCIS-QQNSPKIHEGWWAYKEVVQGSFVPVPSFWGLVNSAWNLCSVGKRQSPVNIETSHMIFDPFLT :  82
CAH10_Cjac : -------MEIVWEVLFLLQANFIVCISAQQNSPKIHEGWWAYKEVVQGSFVPVPSFWGLVNSAWNLCSVGKRQSPVNIETSHMIFDPFLT :  83
CAH10_Ppyg : -------MEIVWEVLFLLQANFIVCISAQQNSPKIHEGWWAYKEVVQGSFVPVPSFWGLVNSAWNLCSVGKRQSPVNIETSHMIFDPFLT :  83
CAH10_Cpor : -------MEIVWEVLFLLQANFIVCISAQQNSPKIHEGWWAYKEVVQGSFVPVPSFWGLVNSAWNLCSVGKRQSPVNIETSHMIFDPFLT :  83
CAH10_Mmul : -------MEIVWEVLFLLQANFIVCISAQQNSPKIHEGWWAYKEVVQGSFVPVPSFWGLVNSAWNLCSVGKRQSPVNIETSHMIFDPFLT :  83
CAH10_Acar : -------MEIVWEVLFLLQANFIVCISAQQNSPKIHEGWWAYKEVVQGSFVPVPSFWGLVNSAWNLCSVGKRQSPVNIETSHMIFDPFLT :  83
CAH10_Ggal : -------MEIVWEVLFLLQANFIVCISAQQNSPKIHEGWWAYKEVVQGSFVPVPSFWGLVNSAWNLCSVGKRQSPVNIETSHMIFDPFLT :  83
CAH10_Tgut : -------MEIVWEVLFLLQANFIVCISAQQNSPKIHEGWWAYKEVVQGSFVPVPSFWGLVNSAWNLCSVGKRQSPVNIETSHMIFDPFLT :  83
CAH10_Dopp : -------MEIVWEVLFLLQANFIVCISAQQNSPKIHEGWWAYKEVVQGSFVPVPSFWGLVNSAWNLCSVGKRQSPVNIETSHMIFDPFLT :  83
CAH10_Mdom : -------MEIVWEVLFLLQANFIVCISAQQNSPKIHEGWWAYKEVVQGSFVPVPSFWGLVNSAWNLCSVGKRQSPVNIETSHMIFDPFLT :  83
CAH10_Mmus : -------MEIVWEVLFLLQANFIVCISAQQNSPKIHEGWWAYKEVVQGSFVPVPSFWGLVNSAWNLCSVGKRQSPVNIETSHMIFDPFLT :  83
CAH10_Ptro : -------MEIVWEVLFLLQANFIVCISAQQNSPKIHEGWWAYKEVVQGSFVPVPSFWGLVNSAWNLCSVGKRQSPVNIETSHMIFDPFLT :  83
CAH10_Mfas : -------MEIVWEVLFLLQANFIVCISAQQNSPKIHEGWWAYKEVVQGSFVPVPSFWGLVNSAWNLCSVGKRQSPVNIETSHMIFDPFLT :  83
CAH10_Cfam : -------MEIVWEVLFLLQANFIVCISAQQNSPKIHEGWWAYKEVVQGSFVPVPSFWGLVNSAWNLCSVGKRQSPVNIETSHMIFDPFLT :  83
CAH10_Ogar : -------MEIVWEVLFLLQANFIVCISAQQNSPKIHEGWWAYKEVVQGSFVPVPSFWGLVNSAWNLCSVGKRQSPVNIETSHMIFDPFLT :  83
CAH10_Pabe : -------MEIVWEVLFLLQANFIVCISAQQNSPKIHEGWWAYKEVVQGSFVPVPSFWGLVNSAWNLCSVGKRQSPVNIETSHMIFDPFLT :  83
CAH10_Hsap : -------MEIVWEVLFLLQANFIVCISAQQNSPKIHEGWWAYKEVVQGSFVPVPSFWGLVNSAWNLCSVGKRQSPVNIETSHMIFDPFLT :  83
CAH10_Ecab : -------MEIVWEVLFLLQANFIVCISAQQNSPKIHEGWWAYKEVVQGSFVPVPSFWGLVNSAWNLCSVGKRQSPVNIETTHMIFDPFLT :  83
CAH10_Dnov : -------MEIVWEVLFLLQANFIVCISAQQNSPKIHEGWWAYKEVVQGSFVPVPSFWGLVNSAWNLCSVGKRQSPVNIETSHMIFDPFLT :  83
CAH10_Btau : -------MEIVWEVLFLLQANFIVCISAQQNSPKIHEGWWAYKEVVQGSFVPVPSFWGLVNSAWNLCSVGKRQSPVNIETSHMIFDPFLT :  83
CAH10_Ocun : ---------------------------AQQNSPKIHEGWWAYKEVVQGSFVPVPSFWGLVNSAWNLCSVGKRQSPVNIETSHMIFDPFLT :  63
CAH10_Ttru : ---------------------------AQQNSPKIHEGWWAYKEVVQGSFVPVPSFWGLVNSAWNLCSVGKRQSPVNIETSHMIFDPFLT :  63
CAH10_Oana : ---------------------------AQQNSPKIHEGWWAYKEVVQGSFVPVPSFWGLVNSAWNLCSVGKRQSPVNIETSHMIFDPFLT :  63
CAH10_Xtro : -------MEIVWEVFFILQASLIVCMSAQQNSPKIHEGWWTYKEVVQGSFVPVPSFWGLVNSAWNLCAVGKRQSPVNIETSHMIFDPFLT :  83
CAH10a_Gac : -------MDIFWEILIILHANVIVCISAQPNPP-----WWAYKEVVQGSFVPVPSFWGLVNSAWNLCSVGKRQSPVNIETSHMIFDPFLQ :  78
CAH10a_Tru : --------------------------------------WWAYKEVVQGSFVPVPSFWGLVNSAWNLCSVGKRQSPVNIETSHMIFDPFLL :  52
CAH10a_Dre : -------MNIIWEIFIILQANLIVCSSAQPNPPKIHEGWWAYKEVVQGSFVPVPSFWGLVNSAWNLCSVGKRQSPVNIETSHMIFDPFLT :  83
CAH10b_Tni : -------MHTVWDIVVILQTFLICTSSAQPITSKLDDSWWAYKDVVQGSFVPVPSFWGLVHSAWNLCAIGKRQSPINIETSHMIFDPYLT :  83
CAH10b_Tru : -------MPNVWDIAVVLQTFIICTSSAQPIASKLDDSWWAYKDVVQGSFVPVPSFWGLVHSAWNLCAIGKRQSPINIETSHMIFDPYLT :  83
CAH10b_Gac : -------MHLVWDLSVILQTFLICTSSAQPLTAKLDAGWWAYKDVVHGSFVPVPSFWGLVHSAWNLCAIGKKQSPIDIDTSRLIFDPYLT :  83
CAH10b_Dre : --------------------------TAQPVSSKLHDGWWAYKEVVQGSFIPVPSFWGLVNTAWNLCAIGKRQSPVNIETSRMIFDPFLN :  64
CAH11_Rnor : -----MGGAARLSAPQALVLWAALGAAAHIGPAPDPEDWWSYKENLQGNFVPGPPFWGLVNAAWSLCSVGKRQSPVDVELKRVLYDPFLP :  85
CAH11_Mmus : -----MGGAARLSAPQALVLWAALGAAAHIGPAPDPEDWWSYKENLQGNFVPGPPFWGLVNAAWSLCAVGKRQSPVDVELKRVLYDPFLP :  85
CAH11_Sscr : -----MGGAARLSAPRALVLWAVLGAAAHIGPAPDPEDWWSYKDNLQGNFVPGPPFWGLVNAAWSLCAVGKRQSPVDVELKRVLYDPFLP :  85
CAH11_Ttru : -----MGGAARLNAPRALLLWAALGAAAHIGPAPDPEDWWSYKDNLQGNFVPGPPFWGLVNAAWSLCAVGKRQSPVDVELKRVLYDPFLP :  85
CAH11_Btau : -----MGAAARLSAPRALVLWAALGAAAHIGPAPDPEDWWSYKDNLQGNFVPGPPFWGLVNAAWSLCAVGKRQSPVDVELKRVLYDPFLP :  85
CAH11_Cfam : -----MGAAARLSAPRALVLWAALGAAAHIGPAPDPEDWWSYKDNLQGNFVPGPPFWGLVNAAWSLCAVGKRQSPVDVELKRVLYDPFLP :  85
CAH11_Mmul : -----MGAAARLSAPRALVLWAALGAAAHIGPAPDPEDWWSYKDNLQGNFVPGPPFWGLVNAAWSLCAVGKRQSPVDVELKRVLYDPFLP :  85
CAH11_Hsap : -----MGAAARLSAPRALVLWAALGAAAHIGPAPDPEDWWSYKDNLQGNFVPGPPFWGLVNAAWSLCAVGKRQSPVDVELKRVLYDPFLP :  85
CAH11_Ptro : -----MGAAARLSAPRALVLWAALGAAAHIGPAPDPEDWWSYKDNLQGNFVPGPPFWGLVNAAWSLCAVGKRQSPVDVELKRVLYDPFLP :  85
CAH11_Pabe : -----MGAAARLSAPRALVLWAALGAAAHIGPAPDPEDWWSYKDNLQGNFVPGPPFWGLVNAAWSLCAVGKRQSPVDVELKRVLYDPFLP :  85
CAH11_Ppyg : -----MGAAARLSAPRALVLWAALGAAAHIGPAPDPEDWWSYKDNLQGNFVPGPPFWGLVNAAWSLCAVGKRQSPVDVELKRVLYDPFLP :  85
CAH11_Cjac : -----MGAAARLSAPRALVLWAALGAAAHIGPAPDPEDWWSYKDNLHGNFVPGPPFWGLVNAAWSLCAVGKRQSPVDVELKRVLYDPFLP :  85
CAH11_Oari : -----MGAAPRLSAPRVLVLWAALGAAAHIGPAPDPEDWWSYKDNLQGNFVPGPPFWGLVNAAWSLCAVGKRQSPVDVDLKRVLYDPFLP :  85
CAH11_Fcat : -----MGVAARLSAPRALVLWAALGAAAHIGPAPDPEDWWSYKDNLQGNFVPGPPFWGLVNAAWSLCAVGKRQSPVDVELKRVLYDPFLP :  85
CAH11_Ecab : -----MGGAAPLSAPQALVLWAALGAAAYIGPAPDPEDWWSYKDNLQGNFVPGPPFWGLVNAAWSLCAVGKRQSPVDVELKRVLYDPFLP :  85
CAH11_Mdom : -----MGGPMRLSPPRALLLGAALGAAAHIGPAPAPEDWWTYKDNLQGSFVPGPPFWGLVNAAWSLCAVGKRQSPVDIELRRVLYDPFLP :  85
CAH11_Cpor : ---------------------------AHIGPAPDPEDWWSYKDNLQGNFVPGPPFWGLVNAAWSLCAVGKRQSPVDVDLKRVLYDPFLP :  63
CAH11_Acar : ------------------------------------EDWWAYKETLHGSFVPGPPFWGLVNSAWSLCAIGKRQSPVDVNTSQMVYDPFLP :  54
CAH11_Xtro : ---------RRVIYP---------GESAQFSPAKTYEEWWAYKEAVQGSFVPGPAFWGLVNSAWNLCTIGKRQSPININTSQIIFDPFLP :  72
CAH10_Blan : ----------MIPYVLLTVALVQQLSTNAEGQAVAWDDWWTYEG------ITGPQFWGLVNSDWVMCKDGKMQSPINFRANQILYDPNLI :  74
CAH8_Rnor  : -------------------MADLSFIEDAVAFPEKEEDEEEEEE-GVEWGYEEGVEWGLVFP----DANGEYQSPINLNSREARYDPSLL :  66
CAH8_Mmus  : -------------------MADLSFIEDAVAFPEKEEDEEEEEEEGVEWGYEEGVEWGLVFP----DANGEYQSPINLNSREARYDPSLL :  67
CAH8_Hsap  : -------------------MADLSFIEDTVAFPEKEEDEEEEEE-GVEWGYEEGVEWGLVFP----DANGEYQSPINLNSREARYDPSLL :  66
CAH8_Mmul  : -------------------MADLSFIEDTVAFPEKEEDEEEEEE-GVEWGYEEGVEWGLVFP----DANGEYQSPINLNSREARYDPSLL :  66
CAH8_Pabe  : -------------------MADLSFIEDTVAFPEKEEDEEEEEE-GVEWGYEEGVEWGLVFP----DANGEYQSPINLNSREARYDPSLL :  66
CAH8_Ptro  : -------------------MADLSFIEDTVAFPEKEEDEEEEEE-GVEWGYEEGVEWGLVFP----DANGEYQSPINLNSREARYDPSLL :  66
CAH8_Cjac  : -------------------MADLSFIEDTVAFPEKEDDEEEEEE-GVEWGYEEGVEWGLVFP----DANGEYQSPINLNSREARYDPSLL :  66
CAH8_Sscr  : -------------------MADLSFIEDSVAFPEKEEDEEEEEEEGVEWGYEEGVEWGLVFP----DANGEYQSPINLNSREARYDPSLL :  67
CAH8_Opri  : -------------------MADLSFIEDSVAFPEKEEDEEEEEE-GVEWGYEEGVEWGLVFP----DANGEYQSPINLNSREARYDPSLL :  66
CAH8_Ppyg  : -------------------MADLSFIEDTVAFPEKEEDEEEEDE-GVEWGYEEGVEWGLVFP----DANGEYQSPINLNSREARYDPSLL :  66
CAH8_Btau  : -------------------MADLSFIEDSVAFPEKEEDEEEEEE-GVEWGYEEGVEWGLVFP----DANGEYQSPINLNSREARYDPSLL :  66
CAH8_Mmur  : ----------------------LSFIEDSVAF-EKEEDEEEEEE-GVEWGYEEGVEWGLVFP----DANGEYQSPINLNSREARYDPSLL :  62
CAH8_Ecab  : -------------------MADLSFIEDSVAFPEKEEDEEEEEE-GVEWGYEEGVEWGLVFP----DANGEYQSPINLNSREARYDPSLL :  66
CAH8_Cpor  : -----------------------------------------------------GVEWGLVFP----DANGEYQSPINLNSREARYDPSLL :  33
CAH8_Mluc  : ------------------------TFPQE------NEQGGEGVES-----TEEGVEWGLVFP----DANGEYQSPINLNSREARYDPSLL :  51
CAH8_Cfam  : -------------------MADLSFIEDSVAFRGYEEGGVLNRKLHQWGWPEGGVEWGLVFP----DANGEYQSPINLNSREARYDPSLL :  67
CAH8_Tsyr  : ------------------------------------------------------VEWGLVFP----DANGEYQSPINLNSREARYDPSLL :  32
CAH8_Ttru  : ------------------------------------------------------VEWGLVFP----DANGEYQSPINLNSREARYDPSLL :  32
CAH8_Oana  : ----------------------LSWGFETLFL---------------------GVEWGLVFP----DANGEYQSPINLNSREARYDPSLL :  43
CAH8_Mdom  : ---------------------SLTFCKTNLSVSELKSDSQPGEK---KWGYEEGVEWGLVFP----DANGEYQSPINLNSREARYDPSLL :  62
CAH8_Ggal  : -------------------MADRSLIEGAEPFPRREEAP--------EWGYEEGVEWGLIFP----DANGEYQSPINLNSREAKYDPSLL :  59
CAH8_Tgut  : ----------------------------------------------------SGVEWGLIFP----DANGEYQSPINLNSREAKYDPSLL :  34
CAH8_Acar  : -----------------------------------------------------GVEWGLIFP----DANGEYQSPINLNSREAKYDPLLL :  33
CAH8_Xtro  : -------------------MADKHYIEDEAVQGEKENQE---------WGYEEGVEWGLLYP----EANGDYQSPINLNSREAMYDPSLL :  58
CAH8_Drer  : -------------------MADN--VIEESGNYP--------GKDELDWGYEEGVEWGLLFP----EANGEYQSPINLNSREARYDPQLL :  57
CAH8_Omyk  : -------------------MADS--VIEESDYYP--------GKDELDWGYQGGVEWGLLFP----DANGEYQSPINLNSREARYDPLLL :  57
CAH8_Gacu  : -------------------MADS--VNEESDYN--------PGKDELAWGYEEGVEWGLHFP----SANGEYQSPINLNSREAEYDPSLL :  57
CAH8_Trub  : -------------------MADS--INEELDYKE-------SGNEELDWGYEEGVEWGLLFP----SANGEYQSPINLNSREAQYDPSLL :  58
CAH8_Olat  : -----------------LCMGGG--VEESLSFLQRRQNDQRLRKDELDWGYEEGVEWGLHFP----AANGEYQSPINLNSREAQYDPSLL :  67
CAH8_Tnig  : -------------------------LVQESWYLK--------GVSPLS-----GVEWGLLFP----SANGEYQSPINLNSREARYDPALL :  48
CAH8_Spur  : MSILKFTKFRKRYLRGKLLTRRSDSREKLIPWVHEDTFDDLLCSLPIKAFSYRGTAEWGIHFQ---EANGDCQSPIDINSREAVYREGLN :  87
CAH8_Blan  : -------------------------------------------------------EWGLHWP----YANGDQQSPVNINSRTATFDETLA :  31
CAH8_Tadh  : ------------------------------------------------MILGPSRWNDYFP----AAKGNRQSPININVDNQHCDGDLK :  37
```

```
                     100         *        120         *        140         *        160         *        180
CAH10_Rnor : PLRINTGGRKVS-GTMYNTGRHVSLRLDKEHLVNISGGPMTYSHRLEEIRLHFGSEDSQGSEHLLNGQAFSGEV-QLIHYNHELYTNVTEA : 172
CAH10_Fcat : PLRINTGGRKVS-GTMYNTGRHVSLRLDKEHLVNISGGPMTYSHRLEEIRLHFGSEDSQGSEHLLNGQAFSGEV-QLIHYNHELYTNVTEA : 171
CAH10_Cjac : PLRINTGGRKVS-GTMYNTGRHVSLRLDKEHLVNISGGPMTYSHRLEEIRLHFGSEDSQGSEHLLNGQAFSGEV-QLIHYNHELYTNVTEA : 172
CAH10_Ppyg : PLRINTGGRKVS-GTMYNTGRHVSLRLDKEHLVNISGGPMTYSHRLEEIRLHFGSEDSQGSEHLLNGQAFSGEV-QLIHYNHELYTNVTEA : 172
CAH10_Cpor : PLRINTGGRKVS-GTMYNTGRHVSLRLDKEHLVNISGGPMTYSHRLEEIRLHFGSEDSQGSEHLLNGQAFSGEV-QLIHYNHELYTNVTEA : 172
CAH10_Mmul : PLRINTGGRKVS-GTMYNTGRHVSLRLDKEHLVNISGGPMTYSHRLEEIRLHFGSEDSQGSEHLLNGQAFSGEV-QLIHYNHELYTNVTEA : 172
CAH10_Acar : PLRINTGGRKVS-GTMYNTGRHVSLRLDKEHLVNISGGPMTYSHRLEEIRLHFGSEDSQGSEHLLNGQAFSGEV-QLIHYNHELYTNVTEA : 172
CAH10_Ggal : PLRINTGGRKVS-GTMYNTGRHVSLRLDKEHLVNISGGPMTYSHRLEEIRLHFGSEDSQGSEHLLNGQAFSGEV-QLIHYNHELYTNVTEA : 172
CAH10_Tgut : PLRLNTGGRKVS-GTMYNTGRHVSLRLDKEHLVNISGGPMTYSHRLEEIRLHFGSEDSQGSEHLLNGQAFSGEV-QLIHYNHELYTNVTEA : 172
CAH10_Dopp : PLRINTGGRKVS-GTMYNTGRHVSLRLDKEHLVNISGGPMTYSHRLEEIRLHFGSEDSQGSEHLLNGQAFSGEV-QLIHYNHELYTNVTEA : 172
CAH10_Mdom : PLRINTGGRKVS-GTMYNTGRHVSLRLDKEHLVNISGGPMTYSHRLEEIRLHFGSEDSQGSEHLLNGQAFSGEV-QLIHYNHELYTNVTEA : 172
CAH10_Mmus : PLRINTGGRKVS-GTMYNTGRHVSLRLDKEHLVNISGGPMTYSHRLEEIRLHFGSEDSQGSEHLLNGQAFSGEV-QLIHYNHELYTNVTEA : 172
CAH10_Ptro : PLRINTGGRKVS-GTMYNTGRHVSLRLDKEHLVNISGGPMTYSHRLEEIRLHFGSEDSQGSEHLLNGQAFSGEV-QLIHYNHELYTNVTEA : 172
CAH10_Mfas : PLRINTGGRKVS-GTMYNTGRHVSLRLDKEHLVNISGGPMTYSHRLEEIRLHFGSEDSQGSEHLLNGQAFSGEV-QLIHYNHELYTNVTEA : 172
CAH10_Cfam : PLRINTGGRKVS-GTMYNTGRHVSLRLDKEHLVNISGGPMTYSHRLEEIRLHFGSEDSQGSEHLLNGQAFSGEV-QLIHYNHELYTNVTEA : 172
CAH10_Ogar : PLRINTGGRKVS-GTMYNTGRHVSLRLDKEHLVNISGGPMTYSHRLEEIRLHFGSEDSQGSEHLLNGQAFSGEV-QLIHYS-ELYTNVTEA : 171
CAH10_Pabe : PLRINTGGRKVS-GTMYNTGRHVSLRLDKEHLVNISGGPMTYSHRLEEIRLHFGSEDSQGSEHLLNGQAFSGEV-QLIHYNHELYTNVTEA : 172
CAH10_Hsap : PLRINTGGRKVS-GTMYNTGRHVSLRLDKEHLVNISGGPMTYSHRLEEIRLHFGSEDSQGSEHLLNGQAFSGEV-QLIHYNHELYTNVTEA : 172
CAH10_Ecab : PLRINTGGRKVS-GTMYNTGRHVSLRLDKEHLVNISGGPMTYSHRLEEIRLHFGSEDSQGSEHLLNGQAFSGEV-QLIHYNHELYTNVTEA : 172
CAH10_Dnov : PLRINTGGRKVS-GTMYNTGRHVSLRLDKEHLVNISGGPMTYSHRLEEIRLHFGSEDSQGSEHLLNGQAFSGEV-QLIHYNHELYTNVTEA : 172
CAH10_Btau : PLRINTGGRKVS-GTMYNTGRHVSLRLDKEHLVNISGGPMTYSHRLEEIRLHFGSEDSQGSEHLLNGQAFSGEV-QLIHYNHELYTNVTEA : 172
CAH10_Ocun : PLRINTGGRKVS-GTMYNTGRHVSLRLDKEHLVNISGGPMTYSHRLEEIRLHFGSEDSQGSEHLLNGQAFSGEV-QLIHYNHELYTNVTEA : 152
CAH10_Ttru : PLRINTGGRKVS-GTMYNTGRHVSLRLDKEHLVNVSGGPMTYSHRLEEVRLHFGSEDSQGSEHLLNGQAFSGEV-QLIHYNHELYTNVTEA : 152
CAH10_Oana : PLRINTGGRKVG-GTMYNTGRHVSLRLDKEQLVNISGGPMTYSHRLEEIRLHFGSEDSQGSEHLLNGQAFSGEV-QLIHYNHELYTNVTEA : 152
CAH10_Xtro : PLRINTGGRKVS-GTMYNTGRHVSLRLDKEHLVNISGGPLTYSHRLEEIRLHFGSEDGQGSEHLLNGQAFSGEV-QLIHYNHELYTNVTDA : 172
CAH10a_Gac : PIKLNTGGRKVG-GTMYNTGRHVSLRLDKEHLVNISGGPMTYSHRLEEIRLHFGSEDGQGSEHLLNGQAFSGEV-QLIHYNHELYTNYTEA : 167
CAH10a_Tru : PIKLNTGGHKVG-GTMYNTGRHVSLRLDKEHLVNISGGPMTYSHRLEEIRLHFGSEDGQGSEHLLNGQAFSGEV-QLIHYNHELYTNYTEA : 141
CAH10a_Dre : PLRLNTGGRKVG-GTMYNTGRHVSLRLDKEHLVNISGGPMTYSHRLEEIRLHFGSEDGQGSEHLLNGQAFSGEV-QLIHYNHELYTNYTEA : 172
CAH10b_Tni : PLKLNTGGRKMG-GTMYNTGKHVSLRPDKAHLVNISGGPLGYSYRLEEVRLHFGSEDSQGSEHLLNGQGFPGEV-QLIHYNQDLYANYTEA : 172
CAH10b_Tru : PLKLNTGGRKMG-GTMYNTGKHVSLRPDKAHLVNISGGPLGYSYRLEEVRLHFGSEDSQGSEHLLNGQGFPGEV-QLIHYNQDLYANYSEA : 172
CAH10b_Gac : PLRLNTAGQKMG-CTMYNTGKHVSLRPDKAHLVNISGGPLGYSYRLEEVRLHFGSEDSQGSEHLLNGQGFTGEV-QLIHYNQDLYGNYTEA : 172
CAH10b_Dre : PLRLNAGQRKVS-GTMYNTGRHVSLRPDKSHLVNISGGPLSYSYRLEEIRLHFGSEDNRGSEHLLNGQAFPGEV-QLIHYNQDLYLNYSDA : 153
CAH11_Rnor : PLRLSTGGEKLR-GTLYNTGRHVSFLPASRPVVNVSGGPLLYSHRLSELRLLFGARDGAGSEHQINHQGFSAEV-QLIHFNQELYGNLSAA : 174
CAH11_Mmus : PLRLSTGGEKLR-GTLYNTGRHVSFLPASRPVVNVSGGPLLYSHRLSELRLLFGARDGAGSEHQINHEGFSAEV-QLIHFNQELYGNLSAA : 174
CAH11_Sscr : PLRLSTGGEKLR-GTLYNTGRHVSFLPAPRPVVNVSGGPLLYSHRLSELLLLFGAHDGAGSEHQINHQGFSAEV-QLIHFNQELYGNLSAA : 174
CAH11_Ttru : PLRLSTGGEKLR-GTLYNTGRHVSFLPAPRPVVNVSGGPLLYSHRLSELRLLFGARDGAGSEHQINHQGFSAEV-QLIHFNQELYGNLSAA : 174
CAH11_Btau : PLRLSTGGEKLR-GTLYNTGRHVSFLPAPRPVVNVSGGPLLYSHRLSELRLLFGARDGAGSEHQINHQGFSAEV-QLIHFNQELYGNLSAA : 174
CAH11_Cfam : PLRLSTGGEKLR-GTLYNTGRHVSFLPAPRPVVNVSGGPLLYSHRLSELRLLFGARDGAGSEHQINHQGFSAEV-QLIHFNQELYGNLSAA : 174
CAH11_Mmul : PLRLSTGGEKLR-GTLYNTGRHVSFLPAPRPVVNVSGGPLLYSHRLSELRLLFGARDGAGSEHQINHQGFSAEV-QLIHFNQELYGNFSAA : 174
CAH11_Hsap : PLRLSTGGEKLR-GTLYNTGRHVSFLPAPRPVVNVSGGPLLYSHRLSELRLLFGARDGAGSEHQINHQGFSAEV-QLIHFNQELYGNFSAA : 174
CAH11_Ptro : PLRLSTGGEKLR-GTLYNTGRHVSFLPAPRPVVNVSGGPLLYSHRLSELRLLFGARDGAGSEHQINHQGFSAEV-QLIHFNQELYGNFSAA : 174
CAH11_Pabe : PLRLSTGGEKLR-GTLYNTGRHVSFLPAPRPVVNVSGGPLLYSHRLSELRLLFGARDGAGSEHQINHQGFSAEV-QLIHFNQELYGNFSAA : 174
CAH11_Ppyg : PLRLSTGGEKLR-GTLYNTGRHVSFLPAPRPVVNVSGGPLLYSHRLSELRLLFGARDGAGSEHQINHQGFSAEV-QLIHFNQELYGNFSAA : 174
CAH11_Cjac : PLRLSTGGEKLR-GTLYNTGRHVSFLPAPRPVVNVSGGPLLYSHRLSELRLLFGARDGAGSEHQINHQGFSAEVVQLIHFNQELYGNFSAA : 175
CAH11_Oari : PLRLSTGGEKLR-GTLYNTGRHVSFLPAPRPVVNVSGGPLLYSHRLSELRLLFGARDGAGSEHQINHQGFSAEV-QLIHFNQELYGNLSAA : 174
CAH11_Fcat : PLRLSTGGEKLR-GTLYNTGRHVSFLPAPRPVVNVSGGPLLYSHRLSELRLLFGARDGAGSEHQINHQGFSAEV-QLIHFNQELYGNLSAA : 174
CAH11_Ecab : PLRLSTGGEKLR-GTLYNTGRHVSFLPAARPVVNVSGGPLLYSHRLSELRLLFGARDGAGSEHQINHQGFSAEV-QLIHFNQELYGNLSAA : 174
CAH11_Mdom : PLRLSTGGEKLR-GTLYNTGRHVSFLPAPRPVVNVSGGPLLYSHRLSELRLLFGARDGAGSEHQINHESFSAEV-QLIHFNQELYGNLSAA : 174
CAH11_Cpor : PLRLSTGGEKLR-GTLYNTGRHVSFLPASRPVVNVSGGPLLYSHRLSELRLLFGARDGAGSEHQINHQGFSAEV-QLIHFNQELYGNLSTA : 152
CAH11_Acar : PLRLSTGGKKIS-GTMYNTGRHVSFRPDPLQLVNVSGGPLLYSHRLQEIRLHFGSEDGFGSEHQIDGETYSAEV-QLIHYNQELYPNISEA : 143
CAH11_Xtro : PLRLSMTGTTVS-GTMHNTGRHVSFRLDKEQPVNISGGPLLYNHRLEEVMLHFGSENSIGSEHLMNKETSAGEV-QLIHYNQDLYSNVTEA : 161
CAH10_Blan : PINFDSDTDKMN-GTLLNTGHDLTFQVDDRNRVNLTGGPLSYLYRLQELRLHYGSIDSQGSEHSVDGYSFPGEL-QLLFYNYDLYGDLAEA : 163
CAH8_Rnor  : DVRLSPNYVVCRDCEVTNDGHTIQVILKSKSVLSGGPLPQGQEFELYEVRFHWGRENQRGSEHTVNFKAFPMEL-HLIHWNSTLFGSIDEA : 156
CAH8_Mmus  : DVRLSPNYVVCRDCEVTNDGHTIQVILKSKSVLSGGPLPQGQEFELYEVRFHWGRENQRGSEHTVNFKAFPMEL-HLIHWNSTLFGSIDEA : 157
CAH8_Hsap  : DVRLSPNYVVCRDCEVTNDGHTIQVILKSKSVLSGGPLPQGHEFELYEVRFHWGRENQRGSEHTVNFKAFPMEL-HLIHWNSTLFGSIDEA : 156
CAH8_Mmul  : DVRLSPNYVVCRDCEVTNDGHTIQVILKSKSVLSGGPLPQGHEFELYEVRFHWGRENQRGSEHTVNFKAFPMEL-HLIHWNSTLFGSIDEA : 156
CAH8_Pabe  : DVRLSPNYVVCRDCEVTNDGHTIQVILKSKSVLSGGPLPQGHEFELYEVRFHWGRENQRGSEHTVNFKAFPMEL-HLIHWNSTLFGSIDEA : 156
CAH8_Ptro  : DVRLSPNYVVCRDCEVTNDGHTIQVILKSKSVLSGGPLPQGHEFELYEVRFHWGRENQRGSEHTVNFKAFPMEL-HLIHWNSTLFGSIDEA : 156
CAH8_Cjac  : DVRLSPNYVVCRDCEVTNDGHTIQVILKSKSVLSGGPLPQGHEFELYEVRFHWGRENQRGSEHTVNFKAFPMEL-HLIHWNSTLFGSIDEA : 156
CAH8_Sscr  : DVRLSPNYVVCRDCEVTNDGHTIQVILKSKSVLSGGPLPQGHEFELYEVRFHWGRENQRGSEHTVNFKAFPMEL-HLIHWNSTLFGSIDEA : 157
CAH8_Opri  : DVRLSPNYVVCRDCEVTNDGHTIQVILKSKSVLSGGPLPQGHEFELYEVRFHWGRENQRGSEHTVNFKAFPMEL-HLIHWNSTLFGSIDEA : 156
CAH8_Ppyg  : DVRLSPNYVVCRDCEVTNDGHTIQVILKSKSVLSGGPLPQGHEFELYEVRFHWGRENQRGSEHTVNFKAFPMEL-HLIHWNSTLFGSIDEA : 156
CAH8_Btau  : DIRLSPNYVVCRDCEVANDGHTIQVILKSKSVLSGGPLPQGHEFELYEVRFHWGRENQRGSEHTVNFKAFPMEL-HLIHWNSTLFGSIDEA : 156
CAH8_Mmur  : DIRLSPNYVVCRDCEVTNDGHTIQVILKSKSVLSGGPLPQGHEFELYEVRFHWGRENQRGSEHTVNFKAFPMEL-HLIHWNSTLFGSIDEA : 152
CAH8_Ecab  : DVRLSPNYVVCRDCEVTNDGHTIQVILKSKSVLLGGPLPQGHEFELYEVRFHWGRENQRGSEHTVNFKAFPMEL-HLIHWNSTLFSSIDEA : 156
CAH8_Cpor  : DVRLSPNYVVCRDCEVTNDGHTIQVILKSKSVLSGGPLPQGHEFELYEVRFHWGRENQRGSEHTVNFKAFPMEL-HLIHWNSTLFGSVDEA : 123
CAH8_Mluc  : DVRLSPNYVVCRDCEVTNDGHTIQVILKSKSVLSGGPLPQGHEFELYEVRFHWGRENQRGSEHTVNFKAFPMEL-HLIHWNSTLFGSIDEA : 141
CAH8_Cfam  : DVRLSPNYVVCRDCEVTNDGHTIQVILKSKSVLSGGPLPQGHEFELYEVRFHWGRENQRGSEHTVNFKAFPMEL-HLIHWNSTLFGSIDEA : 157
CAH8_Tsyr  : DVRLSPNYVVCRDCEVTNDGHTIQVILKSKSVLSGGPLPQGHEFELYEVRFHWGRENQRGSEHTVNFKAFPMEL-HLIHWNSTLFGSIDEA : 122
CAH8_Ttru  : DVRLSPNYVVCRDCEVANDGHAIQVILKSKSVLSGGPLPQGHEFELYEVRFHWGRENQRGSEHTVNFKAFPMEL-HLIHWNSTLFGSIDEA : 122
CAH8_Oana  : EVRLSPNYVVCRDCEVTNDGHTIQIIFKSKSVLAGGPLPRGHEFELHEVRFHWGRENQRGSEHTVNFKAFPMEL-HLIHWNSTLYSSIDEA : 133
CAH8_Mdom  : DIRLSPNYVVCRDCEVTNDGHTIQIVLKSKSVLTGGPLPQGHEFELYEVRFHWGRENQRGSEHTVNFKAFPMEL-HLIHWNSTLYSSIDEA : 152
CAH8_Ggal  : EVRLSPNYVVCRDCEVINDGHSIQIALKSKSVLIGGPLPRGHEFELHDVRFHWGRENQRGSEHTVNFKAFPMEL-HLMHWNSTLYSSIDEA : 149
CAH8_Tgut  : EVRLSPNYVVCRDCEVINDGHSIQIALKSKSVLIGGPLPRGHEFELHDVRFHWGRENQRGSEHTVNFKAFPMEL-HLMHWNSTLYSSIDEA : 124
CAH8_Acar  : EVSLSPNYVVCRDCEVINDGHSVQILLKSKSVLVGGPLPRGHEFELHDVRFHWGRENQRGSEHTVNFKAFPMEL-HLVHWNSTMYHNIDEA : 123
CAH8_Xtro  : EVRLTPSYVVCRDCEVINDGHVVQILLKSKSVLKGGPLPRGHEYELNEVRFHWGKENQRGSEHTVNFKAFPMEL-HLIHWNSTLYRSLEEA : 148
CAH8_Drer  : DVGLNPNYVVCRDCEVINDGHTVRIMLKSKSVVTGGPLPSDHEYELSEVRFHWGKENQRGSEHTVNFKAFPMEL-HLIHWNSTLFNSVEEA : 147
CAH8_Omyk  : DEGLTPNYVVCRDCEVINDGHTVRIMLRSKSVVTGGPLQIDHEYELHEVRFHWGKENQRGSEHTVNFKAFPMEL-HLIHWNTTLFSSVEEA : 147
CAH8_Gacu  : DAGLSPNYVVCRECEVINDGHTVRIILKSKSVVSGGPLPSDHEYELHEVRFHWGKENQRGSEHTVNFKAFPMEL-HLIHWNSTLFNTLEDA : 147
CAH8_Trub  : DVGLSPNYVVCRDCEVINDGHTVRIILKSKSVVTGGPLPSGHEYELHEVRFHWGKENQRGSEHTVNFKAFPMEL-HLIHWNSTLFNTLEDA : 148
CAH8_Olat  : DVGLSPNYVVCRDCEVVNDGHTVRILLKSKSVVTGGPLPSDHEYELHEVRFHWGKENQRGSEHTVNFKAFPMEL-HLIHWNSTLFNSLEDA : 157
CAH8_Tnig  : DVGLAPNYLVCRDCEVLNDGHTVRILLRSKSVVAGGPLPSGHEYELHEVRFHWGKENQRGSEHTVNFKAFPMEL-HLIHWNTTLFSTLEDA : 138
CAH8_Spur  : QPPLEVNYALCRDCDLINTGTGIQIIFKYKPVLSGGPLPQNSKFELAACAFHWGQKDDRGSEHTVNFKAYPMEL-HLIHWNSSTYSTLEEA : 177
CAH8_Blan  : ERPLLISYCLCREWELSNDGHAVQVVPKPKTVTSGGPLPEGHEFELCHLRFHWGREDDRGSEHTVNFKAFPMEL-HLLHWNTSLYSNLADA : 121
CAH8_Tadh  : EDPLDIRYIHSRTNAIKNTGNTIRISFSPGSTIQGGPL--EHQYELLHCSFHWGKTNSRGSEHTVIGKQFPMEV-QLLHWNTSLYKQAEDA : 125
```

```
                         *       200          *       220          *       240          *       260          *
CAH10_Rnor : AKSPNGLVVVSIFIK------VSDSSNPFLNRMLNRDTITRITYKNDAYLLQ-GLNIEELYP-----ETSSFITYDGSMTIPPCYETASWI : 251
CAH10_Fcat : AKSPNGLVVVSIFIK------VSDSSNPFLNRMLNRDTITRITYKNDAYLLQ-GLNIEELYP-----ETSSFITYDGSMTIPPCYETASWI : 250
CAH10_Cjac : AKSPNGLVVVSIFIK------VSDSSNPFLNRMLNRDTITRITYKNDAYLLQ-GLNIEELYP-----ETSSFITYDGSMTIPPCYETASWI : 251
CAH10_Ppyg : AKSPNGLVVVSIFIK------VSDSSNPFLNRMLNRDTITRITYKNDAYLLQ-GLNIEELYP-----ETSSFITYDGSMTIPPCYETASWI : 251
CAH10_Cpor : AKSPNGLVVVSIFIK------VSDSSNPFLNRMLNRDTITRITYKNDAYLLQ-GLNIEELYP-----ETSSFITYDGSMTIPPCYETASWI : 251
CAH10_Mmul : AKSPNGLVVVSIFIK------VSDSSNPFLNRMLNRDTITRITYKNDAYLLQ-GLNIEELYP-----ETSSFITYDGSMTIPPCYETASWI : 251
CAH10_Acar : AKSPNGLVVVSIFMK------VSESSNPFLNRMLNRDTITRITYKNDAYLLQ-GLNIEELYP-----ETASFITYDGSMTIPPCYETASWI : 251
CAH10_Ggal : AKSPNGLVVVSIFMK------VSESSNPFLNRMLNRDTITRITYKNDAYLLQ-GLNIEELYP-----ETSSFITYDGSMTIPPCYETASWI : 251
CAH10_Tgut : AKSPNGLVVVSIFMK------VSESSNPFLNRMLNRDTITRITYKNDAYLLQ-GLNIEELYP-----ETSSFITYDGSMTIPPCYETASWI : 251
CAH10_Dopp : AKSPNGLVVVSIFMK------VSDSSNPFLNRMLNRDTITRITYKNDAYLLQ-GLNIEELYP-----ETSSFITYDGSMTIPPCYETASWI : 251
CAH10_Mdom : AKSPNGLVVVSIFMK------VSDSSNPFLNRMLNRDTITRITYKNDAYLLQ-GLNIEELYP-----ETSSFITYDGSMTIPPCYETASWI : 251
CAH10_Mmus : AKSPNGLVVVSIFIK------VSDSSNPFLNRMLNRDTITRITYKNDAYLLQ-GLNIEELYP-----ETSSFITYDGSMTIPPCYETASWI : 251
CAH10_Ptro : AKSPNGLVVVSIFIK------VSDSSNPFLNRMLNRDTITRITYKNDAYLLQ-GLNIEELYP-----ETSSFITYDGSMTIPPCYETASWI : 251
CAH10_Mfas : AKSPNGLVVVSIFIK------VSDSSNPFLNRMLNRDTITRITYKNDAYLLQ-GLNIEELYP-----ETSSFITYDGSMTIPPCYETASWI : 251
CAH10_Cfam : AKSPNGLVVVSIFIK------VSDSSNPFLNRMLNRDTITRITYKNDAYLLQ-GLNIEELYP-----ETSSFITYDGSMTIPPCYETASWI : 251
CAH10_Ogar : AKSPNGLVVVSIFIK------VSDSSNPFLNRMLNRDTITRITYKNDAYLLQ-GLNIEELYP-----ETSSFITYDGSMTIPPCYETASWI : 250
CAH10_Pabe : AKSPNGLVVVSIFIK------VSDSSNPFLNRMLNRDTITRITYKNDAYLLQ-GLNIEELYP-----ETSSFITYDGSMTIPPCYETASWI : 251
CAH10_Hsap : AKSPNGLVVVSIFIK------VSDSSNPFLNRMLNRDTITRITYKNDAYLLQ-GLNIEELYP-----ETSSFITYDGSMTIPPCYETASWI : 251
CAH10_Ecab : AKSPNGLVVVSIFIK------VSDSSNPFLNRMLNRDTITRITYKNDAYLLQ-GLNIEALYP-----ETSSFITYDGSMTIPPCYETASWI : 251
CAH10_Dnov : AKSPNGLVVVSIFIK------VSDSSNPFLNRMLNRDTITRITYKNDAYLLQ-GLNIEGTYP-----GTSSFINYDESMTIPPCYETAGWI : 251
CAH10_Btau : AKSPNGLVVVSIFIK------VSDSSNPFLNRMLNRDTITRITYKNDAYLLQ-GLNIEELYP-----ETSSFITYDGSMTIPPCYETANWI : 251
CAH10_Ocun : AKSPNGLVVVSIFIK------VSDSSNPFLNRMLNECTITRITYKNDAYLLQ-GLNIEELYP-----ETSSFITYDGSMTIPPCYETASWI : 231
CAH10_Ttru : AKSPNGLVVVSIFIK------VSDSSNPFLNRMLNRDTITRITYKNDAYLLQ-GLNIEELYP-----ETSSFITYDGSMTIPPCYETASWI : 231
CAH10_Oana : AKSPNGLVVVSIFMK------VSDSSNPFLNRMLNRDTITRITYKNDAYLLQ-GLNIEELYP-----ETASFITYDGSMTTPPCYETASWI : 231
CAH10_Xtro : AKSPNGLVVISIFIK------VTDSSNLFLNRMLNRDTITRITYKNDAYLLQ-GLNIEEIYP-----ETSSFITYDGSMTIPPCYETASWI : 251
CAH10a_Gac : AKSPNGLVIVSIFMK------IAETANSFLNRMLNRDTITRITYKNDAYLLT-NLNIEEIYP-----ETISFITYEGSMTIPPCFETATWI : 246
CAH10a_Tru : AKSPNGLVIISIFMK------IAETSNSFLNRMLNRDTITRITYKNDAYLLT-GLNIEEIYP-----DTSSFITYDGSMTIPPCFETVTWI : 220
CAH10a_Dre : AKSPNGLVIVSIFMK------ISETSNSFLNRMLNRDTITRITYKNDAYLLS-GLNIEEVYP-----ETASFITYEGSMTIPPCYETATWI : 251
CAH10b_Tni : AKSPHGIAVVSIFIK------LSENSNSFLNRMLNRDTITRINNRHDAFLLM-GLNIADLYP-----DTTRYITYEGSITIPPCYETSTWI : 251
CAH10b_Tru : AKSPHGIAVVSIFIK------LSENSNSFLNRMLNRDTITRINYRHDAFLLM-GLNIADLYP-----DTTRYITYEGSITIPPCYETSTWI : 251
CAH10b_Gac : AKSPHGIAVVSIFMK------LSENSNAFLNRMLHRDSITRINYKHDAFLLM-GLNIADLYP-----DTTRYITYEGSITVPPCYETSTWI : 251
CAH10b_Dre : VRSPNGIAVVSIFIK------ISEPTNVFLNRMLNRETVTRITYKHDAYLLM-GLNIEELYP-----ETSRFITYEGSITIPPCLETATWI : 232
CAH11_Rnor : SRGPNGLAILSLFVN------VAGSSNPFLSRLLNRDTITRISYKNDAYFLQ-DLSLELLFP-----ESFGFITYQGSLSTPPCSETVTWI : 253
CAH11_Mmus : SRGPNGLAILSLFVN------VAGSSNPFLSRLLNRDTITRISYKNDAYFLQ-DLSLELLFP-----ESFGFITYQGSLSTPPCSETVTWI : 253
CAH11_Sscr : SRGPNGLAILSLFVNV---SQVAGNSNPFLSRLLNRDTITRISYKNDAYFLQ-DLSLELLFP-----ESFGFITYQGSLSTPPCSETVTWI : 256
CAH11_Ttru : SRGPNGLAILSLFVN------VAGSSNPFLSRLLNRDTITRISYKNDAYFLQ-DLSLELLFP-----ESFGFITYQGSLSTPPCSETVTWI : 253
CAH11_Btau : TRGPNGLAILSLFVN------VAGSSNPFLSRLLNRDTITRISYKNDAYFLQ-DLSLELLFP-----ESFGFITYQGSLSTPPCSETVTWI : 253
CAH11_Cfam : SRGPNGLAILSLFVN------VAGSSNPFLSRLLNRDTITRISYKNDAYFLQ-DLSLELLFP-----ESFGFITYQGSLSTPPCSETVTWI : 253
CAH11_Mmul : SRSPNGLAILSLFVN------VAGSSNPFLSRLLNRDTITRISYKNDAYFLQ-DLSLELLFP-----ESFGFITYQGSLSTPPCSETVTWI : 253
CAH11_Hsap : SRGPNGLAILSLFVN------VASTSNPFLSRLLNRDTITRISYKNDAYFLQ-DLSLELLFP-----ESFGFITYQGSLSTPPCSETVTWI : 253
CAH11_Ptro : SRGPNGLAILSLFVN------VASTSNPFLSRLLNRDTITRISYKNDAYFLQ-DLSLELLFP-----ESFGFITYQGSLSTPPCSETVTWI : 253
CAH11_Pabe : SRGPNGLAILSLFVN------VASTSNPFLSRLLNRDTITRISYKNDAYFLQ-DLSLELLFP-----ESFGFITYQGSLSTPPCSETVTWI : 253
CAH11_Ppyg : SRGPNGLAILSLFVN------VASTSNPFLSRLLNRDTITRISYKNDAYFLQ-DLSLELLFP-----ESFGFITYQGSLSTPPCSETVTWI : 253
CAH11_Cjac : SRGPNGLAILSLFVN------VAGSSNPFLSRLLNRDTITRISYKNDAYFLQ-DLSLELLFP-----ESFGFITYQGSLSTPPCSETVTWI : 254
CAH11_Oari : TRGPNGLAILSLFVN------VAGSSNPFLSRLLNRDTITRISYKNDAYFLQ-DLSLELLFP-----ESFGFITYQGSLSTPPCSETVTWI : 253
CAH11_Fcat : SRGPNGLAILSLFVN------VAGSSNPFLSRLLNRDTITRISYKNDAYFLQ-DLSLELLFP-----ESFGFITYQGSLSTPPCSETVTWI : 253
CAH11_Ecab : SRGPNGLAILSLFVN------VAGSSNPFLSRLLNRDTITRISYKNDAYFLQ-DLSLELLFP-----ESFGFITYQGSLSTPPCSETVTWI : 253
CAH11_Mdom : SRGPNGLAVLALFVNQPSPTQVAANSNPFLSRLLNRDTITRISYKNDAYFVQ-DLSLEQLFP-----ESFGFITYQGSMSTPPCSETVTWI : 259
CAH11_Cpor : SRGPNGLAILSLFVN------VAGSSNPFLSRLLNRDTITRISYKNDAYFLQ-DLSLELLFP-----ESFGFITYQGSLSTPPCSETVTWI : 231
CAH11_Acar : SRNPNGLAVISIFAN------IGPSPNPFLTRLLNRETITRISYKNDAYLLH-DLSLEDLYP-----ETFGFITYQGSMTTPPCYETATWI : 222
CAH11_Xtro : SRNPNGLAVISLFLN------VADNSNSFLNRMLNRETITRISFRNEATYIQ-DLSIEDLYP-----DTFGFLTYQGSMTIPPCYETVTWI : 240
CAH10_Blan : TKGTSGLAIISLFIQ------IGNSTNPELDKLLWSDRLQNVTYKGSAARVE-DFNLTNLFP-----TTPHYMTYQGSLTSPDCAETVTWI : 242
CAH8_Rnor  : VGKPHGIVIIALFVQ---------IGKEHVGLKAVTEILQDIQYKGKSKTIP-CFNPNTLLP---DPLLRDYWVYEGSLTIPPCSEGVTWI : 234
CAH8_Mmus  : VGKPHGIAIIALFVQ---------IGKEHVGLKAVTEILQDIQYKGKSKTIP-CFNPNTLLP---DPLLRDYWVYEGSLTIPPCSEGVTWI : 235
CAH8_Hsap  : VGKPHGIAIIALFVQ---------IGKEHVGLKAVTEILQDIQYKGKSKTIP-CFNPNTLLP---DPLLRDYWVYEGSLTIPPCSEGVTWI : 234
CAH8_Mmul  : VGKPHGIAIIALFVQ---------IGKEHVGLKAVTEILQDIQYKGKSKTIP-CFNPNTLLP---DPLLRDYWVYEGSLTIPPCSEGVTWI : 234
CAH8_Pabe  : VGKPHGIAIIALFVQ---------IGKEHVGLKAVTEILQDIQYKGKSKTIP-CFNPNTLLP---DPLLRDYWVYEGSLTIPPCSEGVTWI : 234
CAH8_Ptro  : VGKPHGIAIIALFVQ---------IGKEHVGLKAVTEILQDIQYKGKSKTIP-CFNPNTLLP---DPLLRDYWVYEGSLTIPPCSEGVTWI : 234
CAH8_Cjac  : VGKPHGIAIIALFVQ---------IGKEHVGLKAVTEILQDIQYKGKSKTIP-CFNPNTLLP---DPLLRDYWVYEGSLTIPPCSEGVTWI : 234
CAH8_Sscr  : VGKPHGIAIIALFVQ---------IGKEHVGLKAVTEILQDIQYKGKSKTIP-CFNPNTLLP---DPLLRDYWVYEGSLTIPPCSEGVTWI : 235
CAH8_Opri  : VGKPHGIAIIALFVQ---------IGKEHVGLKAVTEILQDIQYKGKSKTIP-CFNPNTLLP---DPLLRDYWVYEGSLTIPPCSEGVTWI : 234
CAH8_Ppyg  : VGKPHGIAIIALFVQ---------IGKEHVGLKAVTEILQDIQYKGKSKTIP-CFNPNTLLP---DPLLRDYWVYEGSLTIPPCSEGVTWI : 234
CAH8_Btau  : VGKPHGIAIIALFVQ---------IGKEHVGLKAVTEILQDIQYKGKSKTIP-CFNPNTLLP---DPLLRDYWVYEGSLTIPPCSEGVTWI : 234
CAH8_Mmur  : VGKPHGIAIIALFVQ---------IGKEHVGLKAVTEILQDIQYKGKSKTIP-CFNPNTLLP---DPLLRDYWVYEGSLTIPPCSEGVTWI : 230
CAH8_Ecab  : VGKPHGIAIIALFVQ---------IGKEHAGLKAVTEILQDIQYKGKSKTIP-CFNPNTLLP---DPLLRDYWVYEGSLTIPPCSEGVTWI : 234
CAH8_Cpor  : VG-PHGIAIIALFVQ---------IGKEHVGLKAVTEILQDIQYKGKSKTIP-CFNPNTLLP---DPLLRDYWVYDGSLTIPPCSEGVTWI : 200
CAH8_Mluc  : VGKPHGIAIIALFVQ---------IGKEHVGLKAVTEILQDIQYKGKSKIIP-CFNPNTLLP---DPLLRDYWVYEGSLTIPPCSEGVTWI : 219
CAH8_Cfam  : VGKPHGIAIIALFVQ---------IGKEHVGLKAVTEILQDIQYKGKSKTIP-CFNPNTLLP---DPLLRDYWVYEGSLTIPPCSEGVTWI : 235
CAH8_Tsyr  : VGKPHGIAIIALFVQ---------IGKEHVGLKAVTEILQDIQYKGKSKTIP-CFNPNTLLP---DPLLRDYWVYEGSLTIPPCSEGVTWI : 200
CAH8_Ttru  : VGKPRGIAIIALFVQ---------IGKEHVGLKAVTEILQDIQYKGKSKTIP-CFNPNTLLP---DPLLRDYWVYEGSLTIPPCSEGVTWI : 200
CAH8_Oana  : VGKTHGVAIIALFVQ---------IGKEHVGLKAVTDILQDIQYKGKSKTIP-CFNPNTLLP---DPLLRDYWVYEGSLTIPPCSEGVTWI : 211
CAH8_Mdom  : VGKTHGIVIIALFVQ---------IGKEHIGLKAVTDVLQDIQYKGKTKIIP-CFNPNTLLPEKPDPLLRDYWVYEGSLTIPPCSEGVTWI : 233
CAH8_Ggal  : VGKKHGIAIIALFVQ---------IGKEHVGLKAVTEILQDIQYKGKSKTIP-CFNPNSLLP---DPLLRDYWVYEGSLTIPPCSEGVTWI : 227
CAH8_Tgut  : VGKKHGIAIIALFVQ---------IGKEHVGLKAVTEILQDIQYKGKSKTIP-CFNPNSLLP---DPLLRDYWVYEGSLTIPPCSEGVTWI : 202
CAH8_Acar  : IGKKNGIAIIALFVQ---------IGKEHSGLKTVTEILQDIQYKGKSKTIP-CFNPNSLLP---DPLLRDYWVYEGSLTVPPCSEGVTWI : 201
CAH8_Xtro  : MGKVHGIVIISALFVQ--------IGKENIGLKAITEVLQDIFYKGKSKTIP-CFNPNTLLP---DPLLRDYWVYEGSLTMPPCSEGVTWI : 226
CAH8_Drer  : MGKKRGILIIALFVQ---------VGKEHLGLKAITDVLQDLQYKGKTKIIP-CFNPNTLLP---DPLLRDYWVYEGSLTTPPCSENVTWI : 225
CAH8_Omyk  : LGKTNGVLIIALFVQ---------IGKEHLGLKAITEVLQDLQYKGKTKIIP-CFNPNTLLP---DPLLRDYWVYEGSLTTPPCSESVTWI : 225
CAH8_Gacu  : LGKKNGVLTIALFVQ---------IGKEHLGLKAVTEVLQDLQYKGKNKIIP-CFNPNTLLP---DPLLRDYWVYEGSLTTPPCSERVTWI : 225
CAH8_Trub  : LGKKKKSILAQEFVQ---------IGKEHLGLKAITEVLQDLQYKGKNKIIP-CFNPNTLLP---DPLLRDYWVYEGSLTTPPCSEKVTWI : 226
CAH8_Olat  : LGRKNGVLIIALFVQ---------VGKEHLGLKAITEVLQDLQYKGKSKIIP-CFNPNTLLP---DPLLRDYWVYEGSLTTPPCSENVTWI : 235
CAH8_Tnig  : LGQKNGVLIIALFVQ---------IGKEHLGLKAITEVLQDLQYKGKSKIIP-CFNPNTLLP---DPLLRDYWVYEGSLTTPPCSEKVTWI : 216
CAH8_Spur  : MGKPHGISIIALFIQ---------VGREHAGLRTFTDYLEAVQYKGRTLAVTTPFHPSCLLP---DPQLRDFWTYQGSLTTPPCYERVTWI : 256
CAH8_Blan  : MGKRDGIVIMSVIVQ---------IGRENAGVKNFTDHLEDIQYKGKSKTITTAFNPAAFLP---DPALREYWTYEGSLTSPPCYENVQWI : 200
CAH8_Tadh  : MTAPQGLAIMAAFIQ---------IGQENPDLKAITDVITEVRYKDDIREMRLPFNPIHLVP-----RIRDYWTYDGSLTIPPCTENVTWI : 202
```

```
                    280         *        300         *        320         *        340         *        360
CAH10_Rnor : IMNKPVYITRMQ-MHSLRLLSQNQPSQIF------LSMSDNFRPVQPLNNRCIRTNINF---SLQGKDCPNNRAQKLQYRVNEWLLK : 328
CAH10_Fcat : IMNKPVYITRMQ-MS-LRLLSQNQPSQIF------LSMSDNFRPVQPLNNRCIRTNINF---SLQGKDCPNNRAQKLQYRVNEWLLK : 326
CAH10_Cjac : IMNKPVYITRMQ-MHSLRLLSQNQPSQIF------LSMSDNFRPVQPLNNRCIRTNINF---SLQGKDCPNNRAQKLQYRVNEWLLK : 328
CAH10_Ppyg : IMNKPVYITRMQ-MHSLRLLSQNQPSQIF------LSMSDNFRPVQPLNNRCIRTNINF---SLQGKDCPNNRAQKLQYRVNEWLLK : 328
CAH10_Cpor : IMNKPVYITRMQ-MHSLRLLSQNQPSQIF------LSMSDNFRPVQPLNNRCIRTNINF---SLQGKDCPNNRAQKLQYR------- : 321
CAH10_Mmul : IMNKPVYITRMQ-MHSLRLLSQNQPSQIF------LSMSDNFRPVQPLNNRCIRTNINF---SLQGKDCPNNRAQKLQYR------- : 321
CAH10_Acar : IMNKPVYITRMQ-MHSLRLLSQNQPSQIF------LSMSDNFRPVQPLNNRCIRTNINF---SLQGKDCPNNRAQKLQYR------- : 321
CAH10_Ggal : IMNKPVYITRMQ-MHSLRLLSQNQPSQIF------LSMSDNFRPVQPLNNRCIRTNINF---SLQGKDCPNNRAQKLQYRVNEWLLK : 328
CAH10_Tgut : IMNKPVYITRMQ-MHSLRLLSQNQPSQIF------LSMSDNFRPVQPLNNRCIRTNINF---SLQGKDCPNNRAQKLQYRVNEWLLK : 328
CAH10_Dopp : IMNKPVYITRMQ-MHSLRLLSQNQPSQIF------LSMSDNFRPVQPLNNRCIRTNINF---SLQGKDCPNNRAQKLQYR------- : 321
CAH10_Mdom : IMNKPVYITRMQ-MHSLRLLSQNQPSQIF------LSMSDNFRPVQPLNNRCIRTNINF---SLQGKDCPNNRAQKLQYRVNEWLLK : 328
CAH10_Mmus : IMNKPVYITRMQ-MHSLRLLSQNQPSQIF------LSMSDNFRPVQPLNNRCIRTNINF---SLQGKDCPNNRAQKLQYRVNEWLLK : 328
CAH10_Ptro : IMNKPVYITRMQ-MHSLRLLSQNQPSQIF------LSMSDNFRPVQPLNNRCIRTNINF---SLQGKDCPNNRAQKLQYRVNEWLLK : 328
CAH10_Mfas : IMNKPVYITRMQ-MHSLRLLSQNQPSQIF------LSMSDNFRPVQPLNNRCIRTNINF---SLQGKDCPNNRAQKLQYRVNEWLLK : 328
CAH10_Cfam : IMNKPVYITRMQ-MHSLRLLSQNQPSQIF------LSMSDNFRPVQPLNNRCIRTNINF---SLQGKDCPNNRAQKLQYRVNEWLLK : 328
CAH10_Ogar : IMNKPVYITRMQ-MHSLRLLSQNQPSQIF------LSMSDNFRPVQPLNNRCIRTNINF---SLQGKDCPNNRAQKLQYRVNEWLLK : 327
CAH10_Pabe : IMNKPVYITRMQ-MHSLRLLSQNQPSQIF------LSMSDNFRPVQPLNNRCIRTNINF---SLQGKDCPNNRAQKLQYRVNEWLLK : 328
CAH10_Hsap : IMNKPVYITRMQ-MHSLRLLSQNQPSQIF------LSMSDNFRPVQPLNNRCIRTNINF---SLQGKDCPNNRAQKLQYRVNEWLLK : 328
CAH10_Ecab : IMNKPVYITRMQ-MHSLRLLSQNQPSQIF------LSMSDNFRPVQPLNNRCIRTNINF---SLQGKDCPNNRAQKLQYRVNEWLLK : 328
CAH10_Dnov : IMN-PMCITRMQ-IHSLCLLSQNQPSQIV------PNMSDNFRPVRPLNNCCIHPNINF---SLQGKDCPNNRAQELQYRINEWLLK : 327
CAH10_Btau : IMNKPVYITRMQ-MHSLRLLSQNQPSQIF------LSMSDNFRPVQSLNNRCIRTNINF---SLQGKDCPNNRAQKLQYRVNEWLLK : 328
CAH10_Ocun : IMNKPVYITRMQ-MHSLRLLSQNQPSQIF------LSMSDNFRPVQPLNNRCIRTNINF---SLQGKDCPNNRAQKLQYRVNEWLLK : 308
CAH10_Ttru : IMNKPVYITRMQ-MHSLRLLSQNQPSQIF------LSMSDNFRPVQPLNNRCIRTNINF---SLQGKDCPNNRAQKLQYRVNEWLLK : 308
CAH10_Oana : IMNKPVYITRMQ-MHSLRLLSQNQPSQIF------LSMSDNFRPGQPLNNRCIRTNINF---SLQGKDCPNHRPQKLQYRVNEWLLK : 308
CAH10_Xtro : IMNKPIYITRMQ-MHSLRLLSQNQPSQIF------SSMSDNFRPVQSLNNRCIRTNINF---RLQGKDCPNNRAQKLQYK------- : 321
CAH10a_Gac : LMNKPVYLTRMQ-MHSLRLLSQNQPSQIF------LSMSDNVRPVQPLNNRCIRTNINF---SMQGKDCPNNRAQKLQYRVNEWLLK : 323
CAH10a_Tru : LMNKPVYLTRMQ-MHSLRLLSQNLPSQIF------LSMSDNVRPVQPLNNRCIRTNINF---SMQGKDCPNNRAQKLQYRVNEWLLK : 297
CAH10a_Dre : LMNKPIYVTKMQ-MHSMRLLSQNQPSQIF------LSMSDNVRPVQPLNNRCIRTNINF---SMQGKDCPNNRAQKLQYRVNEWLLK : 328
CAH10b_Tni : LINKPVYVTQMQ-MHSLRLLSQNEPYKIF------LSMSDNIRPAQPLLQRCIRTNINF---SKQGRNCPNNRALRPQYRVNQWLLK : 328
CAH10b_Tru : LINKPVYVTQMQ-MHSLRLLSQNEPYKIF------LSMSDNIRPTQPLLQRCIRTNINF---SKQGRNCPNNRALRPQYRVNQWLLK : 328
CAH10b_Gac : LINKPVYVTRMQ-MHSLRLLSQNEPYKIF------LSMSDNTRPPQPLLQRCIRTNINF---NKPGRDCPNNRVLRPQYRVNQWLLK : 328
CAH10b_Dre : LMNKPIYISQIE-MQSLRLLSQNQPSQIF------LSMGDNMRPTQTLHQRCIRTNINF---SQR-RDCPNNRMLRPQYRVNEWLLK : 308
CAH11_Rnor : LIDRALNITSLQ-MHSLRLLSQNPPSQIF------QSLSGNGRPLQPLAHRALRGNRDP---RHPERRCRGPNYRLHVDGGPHGR-- : 328
CAH11_Mmus : LIDRALNITSLQ-MHSLRLLSQNPPSQIF------QSLSGNGRPLQPLAHRALRGNRDP---RHPERRCRGPNYRLHVDGGPHGR-- : 328
CAH11_Sscr : LIDRALNITSLQ-MHSLRLLSQNPPSQIF------QSLSGNGRPLQPLAHRALRGNRDP---RHPERRCRGPNYRLHVDGAPHGR-- : 331
CAH11_Ttru : LIDRALNITSLQ-MHSLRLLSQNPPSQIF------QSLSGNGRPLQPLTHRALRGNRDP---RHPERRCRGPNYRLH---------- : 320
CAH11_Btau : LIDRALNITSLQ-MHSLRLLSQNPPSQIF------QSLSGNGRPLQPLAHRALRGNRDP---RHPERRCRGPNYRLHVDGAPHGR-- : 328
CAH11_Cfam : LIDRALNITSLQ-MHSLRLLSQNPPSQIF------QSLSGNGRPLQPLAHRALRGNRDP---RHPERRCRGPNYRLHVDGAPHGR-- : 328
CAH11_Mmul : LIDRALNITSLQ-MHSLRLLSQNPPSQIF------QSLSGNGRPLQPLAHRALRGNRDP---RHPERRCRGPNYRLHVDGAPHGR-- : 328
CAH11_Hsap : LIDRALNITSLQ-MHSLRLLSQNPPSQIF------QSLSGNSRPLQPLAHRALRGNRDP---RHPERRCRGPNYRLHVDGVPHGR-- : 328
CAH11_Ptro : LIDRALNITSLQ-MHSLRLLSQNPPSQIF------QSLSGNSRPLQPLAHRALRGNRDP---RHPERRCRGPNYRLHVDGVPHGR-- : 328
CAH11_Pabe : LIDRALNITSLQ-MHSLRLLSQNPPSQIF------QSLSGNSRPLQPLAHRALRGNRDP---RHPERRCRGPNYRLHVDGAPHGR-- : 328
CAH11_Ppyg : LIDRALNITSLQ-MHSLRLLSQNPPSQIF------QSLSGNSRPLQPLAHRALRGNRDP---RHPERRCRGPNYRLHVDGAPHGR-- : 328
CAH11_Cjac : LIDRALNITSLQ-MHSLRLLSQNPPSQIF------QSLSGNGRPLQPLAHRALRGNRDP---RHPERRCRGPNYRLH---------- : 321
CAH11_Oari : LIDRALNITSLQ-MHSLRLLSQNPPSQIF------QSLSGNGRPLQPLAHRALRGNRDP---RHPERRCRGPNYRLHVDDVPHGL-- : 328
CAH11_Fcat : LIDRALNITSLQ---------------------------------------------------------------------------- : 265
CAH11_Ecab : LIDRALNITSLQVILCLHHLSALPP--------------------------------------------------------------- : 278
CAH11_Mdom : LIDRALNVTSLQ-MHSLRLLSQNPPSQIF------QSLSGNGRPLQPLAHRALRGNRDS---RHPERRCRGPNYRLH--GMPWSL-- : 332
CAH11_Cpor : LIDRPLNITSLQ-MHSLRLLSQNPPSQIF------QSLSGNGRPLQPLAHRALRGNRDP---RHPERRCRGPNYRLH---------- : 298
CAH11_Acar : LIDRPINITSVQ-IHSLRLLSQNLPSQIF------RSMSDNSRPLQPLLHRSLRGNRDP---RRPTRRCKGGNFRLH---------- : 289
CAH11_Xtro : VIDRPLNITSMQ-MHSLRLLSQNQPSQIF------QSMSDNFRPVQPLFHRGLRGNLDF---RKPGRKCKGANYKLHIDRPTSSKNA : 317
CAH10_Blan : VMNKPVYISRKK-MNQLRNLWQNTMGQKE------TPLADNFRPTSNLNSRTIRTNINFTTKDKMGKPCPTMKSKKAYQSSNRC--- : 319
CAH8_Rnor  : LFRYPLTISQLQ-IEEFRRLRTHVKGAEL-VEGCDGILGDNFRPTQPLSDRVIRAFSSQRNLKQAHLHQGRRRWSHRVL-------- : 311
CAH8_Mmus  : LFRYPLTISQMQ-IEEFRRLRTHVKGAEL-VEGCDGILGDNFRPTQPLSDRVIRAAFQ----------------------------- : 291
CAH8_Hsap  : LFRYPLTISQLQ-IEEFRRLRTHVKGAEL-VEGCDGILGDNFRPTQPLSDRVIRAAFQ----------------------------- : 290
CAH8_Mmul  : LFRYPLTISQLQ-IEEFRRLRTHVKGAEL-VEGCDGILGDNFRPTQPLSDRVIRAAFQ----------------------------- : 290
CAH8_Pabe  : LFRYPLTISQLQ-IEEFRRLRTHVKGAEL-VEGCDGILGDNFRPTQPLSDRVIRAAFQ----------------------------- : 290
CAH8_Ptro  : LFRYPLTISQLQ-IEEFRRLRTHVKGAEL-VEGCDGILGDNFRPTQPLSDRVIRAAFQ----------------------------- : 290
CAH8_Cjac  : LFRYPLTISQLQ-IEEFRRLRTHVKGAEL-VEGCDGILGDNFRPTQPLSDRVIRAAFQ----------------------------- : 290
CAH8_Sscr  : LFRYPLTISQLQ-IEEFRRLRTHVKGAEL-VEGCDGILGDNFRPTQPLSDRVIRAAFQ----------------------------- : 291
CAH8_Opri  : LFRYPLTISQLQ-IEEFRRLRTHVKGAEL-VEGCDGILGDNFRPTQPLSDRVIRAAFQ----------------------------- : 290
CAH8_Ppyg  : LFRYPLTISQLQ-IEEFRRLRTHVKGAEL-VEGCDGILGDNFRPTQPLSDRVIRAAFQ----------------------------- : 290
CAH8_Btau  : LFRYPLTISQLQ-IEEFRRLRTHVKGAEL-VEGCDGILGDNFRPTQPLSDRVIRAAFQ----------------------------- : 290
CAH8_Mmur  : LFRYPLTISQLQ-IEEFRRLRTHVKGAEL-VEGCDGILGDNFRPTQPLSDRVIRAAFQ----------------------------- : 286
CAH8_Ecab  : LFRYPLTISQLQ-IEEFRRLRTHVKGAEL-VEGCDGILGDNFRPTQPLSDRVIRAAFQ----------------------------- : 290
CAH8_Cpor  : LFRYPLTISQLQ-IEEFRRLRTHVKGAEL-VEGCDGILGDNFRPTQPLSDRVIRAAFQ----------------------------- : 256
CAH8_Mluc  : LFRYPLTISQLQ-IEEFRRLRTHVKGAEL-VEGCDGILGDNFRPTQPLSDRVIRAAFQ----------------------------- : 275
CAH8_Cfam  : LFRYPLTISQLQ-IEEFRRLRTHVKGAEL-VEGCDGILGDNFRPTQPLSDRVIRAAFQ----------------------------- : 291
CAH8_Tsyr  : LFRYPLTISQLQ-IEEFRRLRTHVKGAEL-VEGCDGILGDNFRPTQPLSDRVIRAAFQ----------------------------- : 256
CAH8_Ttru  : LFRYPLTISQLQ-IEEFRRLRTHAKGAEL-VEGCDGILGDNFRPTQPLSDRVIRAAFQ----------------------------- : 256
CAH8_Oana  : LFRYPLTISQIQ-IEEFRRLRTHIKGAEL-VEGCDGILGDNFRPTQPLSDRVIRAAFQ----------------------------- : 267
CAH8_Mdom  : LFRYPLTISQIQ-IEEFRRLRTHVKGAEL-LEGCDGILGDNFRPTQPLSDRVIRAAFQ----------------------------- : 289
CAH8_Ggal  : LFRYPLTVSQVQ-IEEFRRLRTHVKGAEL-LEGCDGILGDNFRPTQPLSDRVIRAAFQ----------------------------- : 283
CAH8_Tgut  : LFRYPLTVSQVQ-IEEFRRLRTHVKGAEL-LEGSDGILGDNFRPTQPLSDRVIRAAFQ----------------------------- : 258
CAH8_Acar  : LFRYPLTVSQLQ-IEEFRRLRTHVKGTEL-LDSCDGILGDNFRPTQPLSDRIIRAAFQ----------------------------- : 257
CAH8_Xtro  : LFRYPLTVSQTQ-IEEFRRLRTHIKGADL-PDGCDGLMADNFRPTQPLSDRIIRAAFQ----------------------------- : 282
CAH8_Drer  : LYRYPLTISQMQ-IEEFRRLRPHVKGAEL-PEGNDGMLGDNFRPTQPLSDRVVRAAFQ----------------------------- : 281
CAH8_Omyk  : LYRYPLTISQLQ-IEEFRRLRSHLKGADL-PEGNDGMLGDNFRPSQPLSDRTVRAAFQ----------------------------- : 281
CAH8_Gacu  : LYRYPLTISQSQ-IEEFRRLRSHVKGAEL-LEGNDGMLGDNFRPTQPLSDRTVRAAFQ----------------------------- : 281
CAH8_Trub  : LFRYPLTISQIQ-IEEFRRLRSHVKGAEL-LEGNDGLLGDNFRPTQPLSDRTVRAAFQ----------------------------- : 282
CAH8_Olat  : LYRYPLTISQMQ-IEEFRRLRSHVKGAEL-LEGNDGMLGDNFRPTQPLSDRTVRAAFQ----------------------------- : 291
CAH8_Tnig  : LFRYPLTISQVQ-IEEFRRLRSHIKGAEL-LEGNDGLLGDNFRPTQPLSDRMVRAAFQ----------------------------- : 272
CAH8_Spur  : LLRYPLTISISQ-MEEFRRLKNHVKGDFS-FRGEEGLLVDNFRPTQPLNDRTIEASFQ----------------------------- : 312
CAH8_Blan  : IFRYPLTISHVQ-MEEFRRLRCHAKGQPP-PPGEEGMLCDNFRPTQPIKSRVIRASFQ----------------------------- : 256
CAH8_Tadh  : IFRYPLSISESQ-IQGYRSLRAFFRQDRQDVASREGALFDNFRPVQSMNNRPLRASFEIDVFSGLLKNIPQWKLGNVIPKDSSDSKL : 288
```
